# Prognosis and therapeutic significance of IGF-1R-related signaling pathway gene signature in glioma

**DOI:** 10.3389/fcell.2024.1375030

**Published:** 2024-04-11

**Authors:** Zhen Liu, Liangwang Yang, Wenqi Wu, Zejun Chen, Zhengxing Xie, Daoming Shi, Ning Cai, Shenghua Zhuo

**Affiliations:** ^1^ Department of Neurosurgery, Affiliated Hospital of Jiangsu University, Zhenjiang, Jiangsu, China; ^2^ Department of Neurosurgery, First Affiliated Hospital of Hainan Medical University, Haikou, Hainan, China; ^3^ Department of Neurology, Affiliated Hospital of Jiangsu University, Zhenjiang, Jiangsu, China; ^4^ Department of General Surgery, Affiliated Hospital of Jiangsu University, Zhenjiang, Jiangsu, China

**Keywords:** glioma, IGF-1R signaling, tumor immune microenvironment, immunotherapy/chemotherapy response, HSP90B1

## Abstract

**Background:**

Glioma is the most common cancer of the central nervous system with poor therapeutic response and clinical prognosis. Insulin-like growth factor 1 receptor (*IGF-1R*) signaling is implicated in tumor development and progression and induces apoptosis of cancer cells following functional inhibition. However, the relationship between the *IGF-1R*-related signaling pathway genes and glioma prognosis or immunotherapy/chemotherapy is poorly understood.

**Methods:**

LASSO–Cox regression was employed to develop a 16-gene risk signature in the TCGA-GBMLGG cohort, and all patients with glioma were divided into low-risk and high-risk subgroups. The relationships between the risk signature and the tumor immune microenvironment (TIME), immunotherapy response, and chemotherapy response were then analyzed. Immunohistochemistry was used to evaluate the HSP90B1 level in clinical glioma tissue.

**Results:**

The gene risk signature yielded superior predictive efficacy in prognosis (5-year area under the curve: 0.875) and can therefore serve as an independent prognostic indicator in patients with glioma. The high-risk subgroup exhibited abundant immune infltration and elevated immune checkpoint gene expression within the TIME. Subsequent analysis revealed that patients in the high-risk subgroup benefited more from chemotherapy. Immunohistochemical analysis confirmed that HSP90B1 was overexpressed in glioma, with significantly higher levels observed in glioblastoma than in astrocytoma or oligodendrocytoma.

**Conclusion:**

The newly identified 16-gene risk signature demonstrates a robust predictive capacity for glioma prognosis and plays a pivotal role in the TIME, thereby offering valuable insights for the exploration of novel biomarkers and targeted therapeutics.

## 1 Introduction

Glioma is the most aggressive primary malignant tumor of the central nervous system (CNS) and accounts for 81% of intracranial malignancies (Ostrom et al., 2019). Among gliomas, glioblastoma (GBM) represents the predominant high-grade subtype (Louis et al., 2021), comprising 49.1% of all CNS malignancies (Ostrom et al., 2021). GBM is characterized by a low survival rate (5-year survival rate: 6.8%) (Ostrom et al., 2021) and exhibits extreme malignancy and invasiveness (Louis et al., 2021). Because of the high proliferation rate, cellular heterogeneity, and extensive infltration capacity of gliomas, treatment response remains poor for these patients (DeCordova et al., 2020). Recently, several molecular biomarkers have been identified for improved diagnosis, treatment selection, and prognostic assessment of glioma (Hu and Qu, 2021; Zhuo et al., 2023).

The insulin-like growth factor 1 receptor (*IGF-1R*), a member of the receptor tyrosine kinase family, remains unmutated in cancers (Baserga, 1995) and exhibits high expression in various tumors (Soni et al., 2023), thus establishing itself as one of the most extensively investigated kinase targets. The correlation between elevated levels of *IGF-1R* pathway signaling and cancer is significantly positive (Baserga, 1995). And the inhibition of *IGF-1R* itself or the downstream signal transduction cascade, namely, the PI3K/AKT/MTOR pathway, exerts potent stimulation on autophagy (Troncoso et al., 2012; Galluzzi et al., 2014) and other adaptive mechanisms (Wang et al., 2020) to achieve anti-tumor efficacy. Therefore, it is considered a crucial target for the development of anti-cancer therapeutics. However, targeting it has often produced the disappointing outcomes arising from the intricate crosstalk with numerous downstream signaling pathways. Despite continuous advancements in inhibitors targeting this specific receptor (Wu et al., 2021), no anticancer drugs have received approval from the Food and Drug Administration (FDA) to date. This suggests that the *IGF-1R* signaling pathway is complex and its pathophysiological role remains incompletely elucidated. Consequently, it is imperative to further investigate the activation mechanism of *IGF-1R* and its downstream signaling pathway in order to identify key molecules driving tumor proliferation.

The relationship between gliomas and *IGF-1R* has been previously reported inhibition of *IGF-1R* can effectively suppress the growth of GBM cells either by directly reducing tumor cell proliferation or through indirect antiangiogenic effects (Zamykal et al., 2015). The study conducted by Tan et al. demonstrates that in glioma, the dual inhibition of *IGF-1R* and *STAT3* sensitizes *STAT3*-low cells and improves the survival of orthotopic xenograft model mouse and concluded that *STAT3*-mediated expression features can be used for precise treatment of patients (Tan et al., 2019). Wei et al. clearly showed that the interaction between *IGF-1R* by *CXCL14* secreted by tumor cells, initiates the subsequent activation of *IGF-1R* and its downstream mediators, *ERK* and *AKT*, involved in the protumorigenic effects for glioblastoma (Wei et al., 2023). Nevertheless, the prognostic and therapeutic significance of *IGF-1R*-related signaling pathway genes (IGFIRS) in gliomas has not been investigated. The independent prognostic value of IGF1RS in glioma, its relationship with the immune microenvironment, and its efficacy in predicting treatment response remain to be investigated.

Therefore, in this study, we initially identified *IGF-1R*-related signaling pathway genes with prognostic value in patients with glioma by analyzing multiple public cohorts and their corresponding survival information. LASSO–Cox regression analysis was used to establish and assess IGF1RS as well as explore its association with the clinicopathological characteristics, prognosis, tumor immune microenvironment (TIME), and therapy response of glioma. Moreover, the marker heat shock protein 90β family member 1 (*HSP90B1*) was screened from the genes included in IGF1RS and its expression was verified in cell lines and clinical glioma specimens. This study provides the basis for further exploration of enhanced personalized treatment options for patients with glioma using *IGF-1R*-related pathway genes.

## 2 Materials and methods

### 2.1 Data collection

The Chinese Glioma Genome Atlas (CGGA, http://www.cgga.org.cn/index.jsp) RNAseq data (mRNAseq_325 [CGGA325] and CGGA mRNAseq_693 [CGGA693]), along with the corresponding clinical and molecular information (gender, age, overall survival [OS], survival state, World Health Organization grade, isocitrate dehydrogenase [*IDH*] mutation status, and 1p/19q codeletion status) were obtained from CGGA in-house data. Similarly, the Tumor Genome Atlas (TCGA) RNAseq data (TCGA-GBMLGG) and its matching information were downloaded from other data in CGGA other data. Primary cases were retained, and patients with missing survival data were excluded. In total, 110 *IGF-1R*-related signaling pathway genes were extracted from the “Development IGF-1 receptor signaling SuperPath” module in PathCards (https://pathcards.genecards.org).

### 2.2 Screening of *IGF-1R*-related signaling pathway genes and calculation of IGF1RS

The relationship between OS and gene expression levels was assessed using univariate Cox regression analysis. Cox regression analyses were conducted using the Survival (3.4–0) package in R software (version 4.1.3), and hazard ratios (HR) along with their corresponding 95% confidence intervals (95% CI) were calculated. In all cohorts, genes with *p* values <0.05 were considered to have significant prognostic potential and were retained. Subsequently, the least absolute shrinkage and selection operator (LASSO)–Cox regression models were used to screen the optimum risk genes for analysis and to establish a prognostic risk model in TCGA-GBMLGG. The penalty parameter (λ) of the model was determined through ten-fold cross-validation, selecting the value of λ that yielded the lowest partial likelihood deviance. In addition, the IGF1RS of each patient was calculated based on the expression level of the genes and their corresponding coefficients derived from LASSO–Cox regression. IGF1RS was calculated using the following formula:

$$\text{riskScore}=\sum_{i}^{16}\text{Xi}*\text{Yi }\left(X:\text{coefficients},Y:\text{gene expression level}\right)$$

### 2.3 Survival analysis

Based on the median IGF1RS, patients were divided into low-risk and high-risk subgroups. Then, a t-distributed stochastic neighbor embedding (t-SNE)-based approach was adopted to explore the low-risk and high-risk assignments. Receiver operating characteristic (ROC) curve, Kaplan–Meier (K–M) survival curve, ranked dot plots, and scatter plots were used to assess the efficiency of IGF1RS in predicting survival.

### 2.4 Development of a prognostic nomogram

Univariate and multivariate Cox regression analyses were used to screen independent prognostic prediction biomarkers to construct a nomogram in TCGA-GBMLGG, and the HRs along with their corresponding 95% CI were calculated. The prognostic effect of the model was evaluated using the concordance index (C index). The consistency between the predicted and actual survival was demonstrated by 1-, 3-, and 5-year prediction calibration curves. In addition, ROC and decision curve analysis (DCA) curves, including nomogram score, IGF1RS, and age, were plotted to further evaluate the model’s performance.

### 2.5 Functional enrichment analysis of IGF1RS

The R packages “clusterProfiler” (v.4.8.1) and “ReactomePA” (v.1.44.0) were used to visualize the top six results of the Gene Ontology (GO) and Reactome pathways for the 16 risk genes.

### 2.6 Immune infltration analysis

The ESTIMATE algorithm was used to evaluate tumor purity and tumor immune score (includes ImmueScore, StromalScore, and EstimateScore). The microenvironment cell population (MCP) algorithm was employed to calculate eight immune cell populations and two stromal cell populations in each sample. In addition, the extent of immune infltration in the gliomas was evaluated using single-sample gene set enrichment analysis (ssGSEA).

### 2.7 Prediction of immunotherapy/chemotherapy response

The Submap algorithm (Hoshida et al., 2007) was employed to predict the clinical response to PD1 and CTLA4 immune checkpoint blocking within the low-risk and high-risk groups. The Genomics of Drug Sensitivity in Cancer (GDSC) database was used to predict the chemotherapeutic response. The prediction process was conducted using the R package “pRRophetic” (version 0.5), where ridge regression was employed to estimate the half-maximal inhibitory concentration (IC50) of each sample. Subsequently, the GDSC website (https://www.cancerrxgene.org/) was used to identify the target pathways of drugs exhibiting significant differences in high- and low-risk groups, while displaying the correlation coefficient of drugs based on the target pathways. The Connectivity Map (cMap) is a comprehensive database that contains over 6,900 expression profiles and 1,309 drugs, which enables the prediction of molecular compounds for diseases by calculating the connectivity between the gene-expression signatures of the patient and all the gene-expression signatures in the database (Lamb et al., 2006). This resource was utilized for drugs validation by inputting the differentially expressed genes, which were identified using the “limma” package (version 3.56.2) (Ritchie et al., 2015), that exhibited upregulation or downregulation between low-risk and high-risk subgroups of IGF1RS (|log2 Fold Change| > 0.3 and *P*-adjusted values <0.05).

### 2.8 HSP90B1 expression, cell culture, and Western blot analyses

Using the GEPIA website (http://gepia.cancer-pku.cn/) (Tang et al., 2017), we analyzed the differences in *HSP90B1* mRNA expression among normal brain tissues, low-grade glioma, and GBM. The UALCAN database (http://ualcan.path.uab.edu/) (Chandrashekar et al., 2017) was used to identify variations in HSP90B1 protein levels between GBM and normal brain tissues.

Human normal astrocytes (HA 1800) and a malignant glioma cell line (U118MG) were purchased from Jennio Biotech (Guangzhou, China). T98G, U251, and LN229 cells were purchased from Procell Life Science and Technology (Wuhan, China). The cells were authenticated using short tandem repeat profiling and screened to exclude *mycoplasma* contamination. Cells were cultured in high-glucose Dulbecco’s modified Eagle’s medium supplemented with 10% fetal bovine serum and 1% penicillin–streptomycin (15140-122, Gibco) and incubated in a humidified CO_2_ incubator at 37°C. The medium was replaced two to three times a week. The aforementioned cells were lysed using RIPA buffer. Then, 20 μg protein, quantified using the BCA kit, was subjected to 10% sodium dodecyl sulfate polyacrylamide gel electrophoresis (SDS-PAGE) and transferred onto a polyvinylidene fluoride membrane. The membrane was blocked with 5% skim milk for 1 h and then incubated with diluted primary antibodies (HSP90B1: 1:10000, 14700-1-AP, proteintech, Wuhan, China; Actin: 1:15000, GB15003-100, Servicebio, Wuhan, China) overnight at 4°C. Subsequently, the membrane was hybridized with a secondary antibody. The expression levels of the proteins were detected using an enhanced chemiluminescence assay.

### 2.9 Immunofluorescence, single-cell expression, and immunohistochemistry analyses

Immunofluorescence images depicting HSP90B1 distribution in the glioma cell line U251 were downloaded from the Human Protein Atlas (HPA) database (https://www.proteinatlas.org/) (Uhlén et al., 2015). Four 10X genomics-based single-cell sequencing GBM cases in the CHARTS (https://charts.morgridge.org/) (Bernstein et al., 2021) database and the uniform manifold approximation and projection dimensionality reduction method were used to examine the predicted cell type composition (Zhuo et al., 2023) and *HSP90B1* expression.

HSP90B1 expression was detected in 101 glioma tissues diagnosed by pathologists collected from the First Affiliated Hospital of Hainan Medical University. The studies involving human participants were reviewed and approved by the Humanities Ethics Committee of the First Affiliated Hospital of Hainan Medical University (Ethics Approval Number: 2023-KYL-124). All research procedures adhered to the code of ethics of the institution, the National Research Council, and the 1975 Declaration of Helsinki and its subsequent amendments. Informed consent was obtained from all participants prior to their inclusion in the study.

Paraffin-embedded glioma tissue sections, with a thickness of 5 μm, were blocked with 5% BSA (Sigma, B2064) for 20 min and subsequently incubated overnight at 4 °C with primary polyclonal anti-HSP90B1 (14700-1-AP, proteintech, 1:200). After washing with phosphate buffered saline, the sections were incubated at 37°C for 30 min with biotinylated immunoglobulin G (IgG) secondary antibodies (diluted to a concentration of 1:200). Dako REAL™ EnVision™ detection was used for executing the system secondary antibody and diaminobenzidine color development. Scanned images of stained sections were captured using a digital pathology slide scanner (KFBIO KF-PRO-120), and graphical representation was performed using K-Viewer software (version 1.5.5.6). The assessment of the results was independently conducted by two individuals primarily based on the staining intensity and count of positive cells. Cell scores for staining ranging from 0% to 25% were denoted as (+, 1), cells exhibiting staining between 26% and 50% were assigned a score of (++, 2), cells with staining in the range of 51%–75% were given a score of (++, 3); and cells displaying staining within the range of 76%–100% were assigned a score of (++++, 4). The staining color was assessed as light-yellow particles (+, 1), brown-yellow particles (++, 2), or brown particles (+++, 3). The final score was determined by multiplying the staining number score by the staining color score.

### 2.10 Statistical analysis

The analyses in this study were performed using R software 4.1.3. Wilcoxon’s rank sum test was used to compare the differences in gene expression between the two groups, while the Kruskal–Wallis test was employed to analyze the differences among the three groups. The Chi-square test was used to compare the clinical data between patients in the low-risk and high-risk groups. Log-rank tests and Kaplan–Meier (K–M) plots were used to compare the survival rates of the low-risk and high-risk groups. Spearman’s correlation coefficient was used to conduct correlation analyses. A significance level of *p* < 0.05 was considered statistically significant.

## 3 Results

### 3.1 Identification of IGF-1R-related signaling pathway genes with independent prognostic value in glioma and establishment of IGF1RS

**FIGURE 1:**
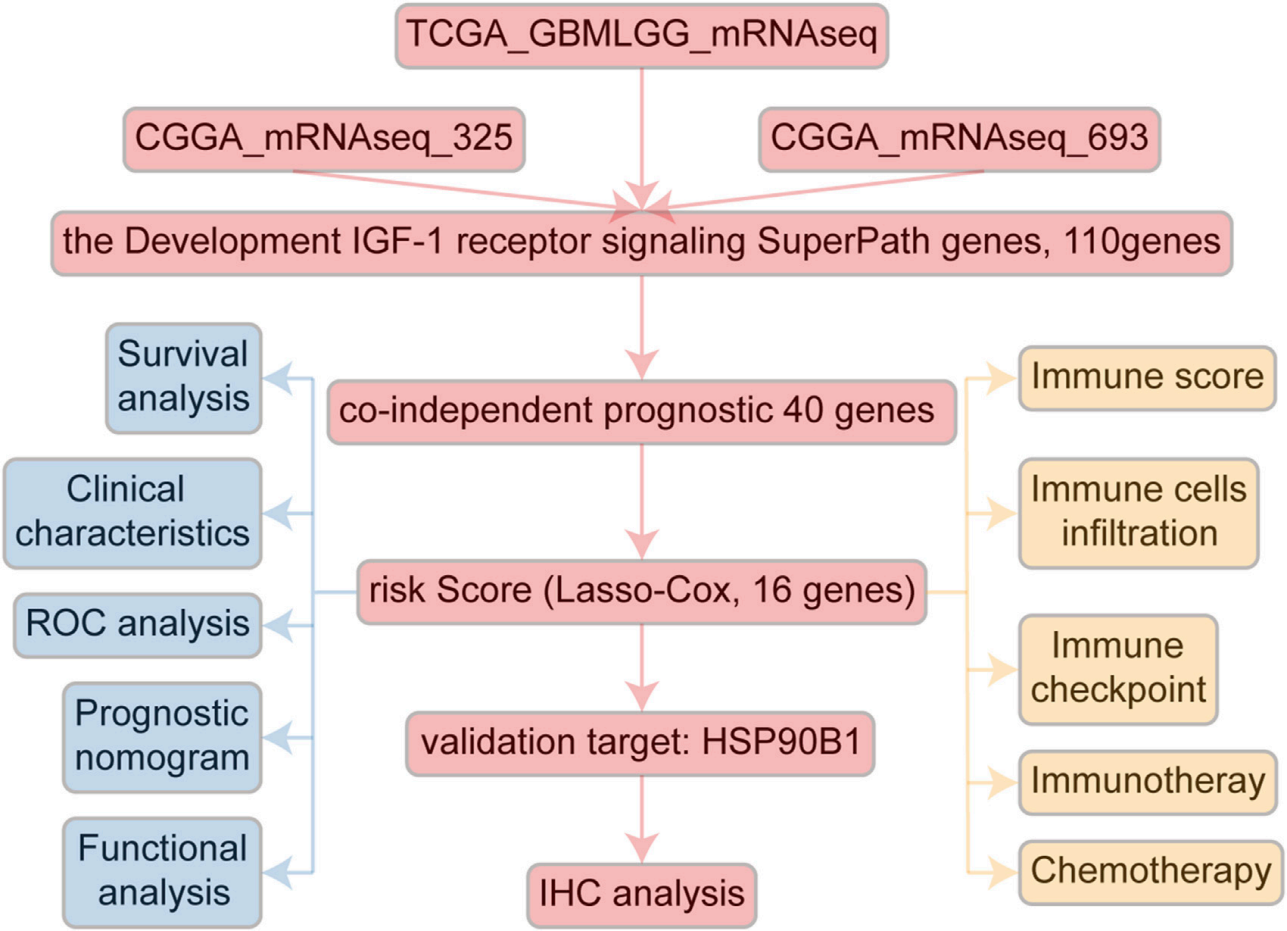
The flowchart of this study.

**FIGURE 2:**
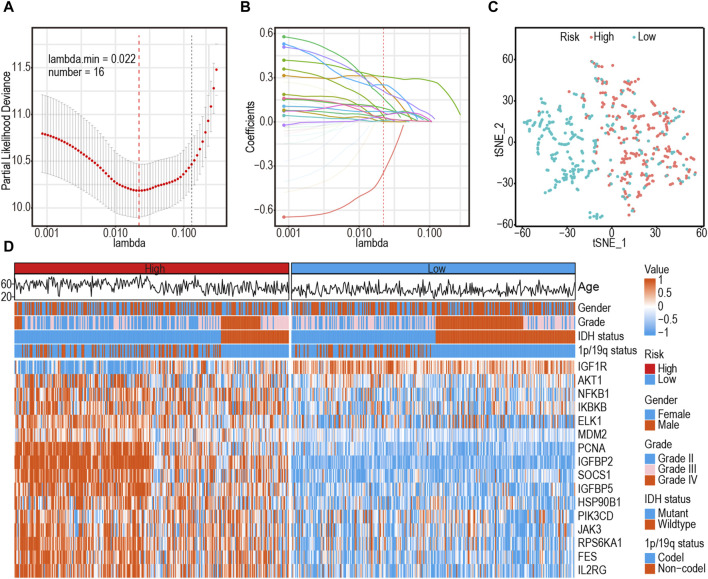
Identification of a 16-gene risk signature for overall survival by LASSO–Cox regression analysis in TCGA-GBMLGG cohort. **(A)** Cross-validation for tuning parameter selection in the proportional hazards model. **(B)** The coefficient spectrum of 16-gene in gliomas. **(C)** t-SNE analysis supported the stratification into low- and high-risk subgroups. **(D)** Heatmap shows the association between risk and clinicopathological features based on the 16-gene risk signature. LASSO: least absolute shrinkage and selection operator.

A comprehensive flow chart of this article is illustrated in Figure 1. Forty IGF-1R-related signaling pathway genes were screened via univariate Cox regression analysis in the TCGA-GBMLGG, CGGA325, and CCGA693 cohorts. Subsequently, the LASSO–Cox algorithm was used to screen 16 genes (*AKT1*, *IGFBP5*, *IGF1R*, *JAK3*, *PIK3CD*, *RPS6KA1*, *IKBKB*, *MDM2*, *SOCS1*, *NFKB1*, *ELK1*, *PCNA*, *IL2RG*, *IGFBP2*, *FES*, and *HSP90B1*) in TCGA-GBMLGG (Figures 2A, B). The HR and 95% CI of 16 genes are shown in Supplementary Figure S1. IGF1RS was calculated by multiplying the expression level of the genes by their corresponding regression coefficients (IGF1RS = −0.344009920 × expression level of *AKT1*+ 0.093474674 × expression level of *IGFBP5* + 0.039986077 × expression level of *IGF1R* + 0.010017526 × expression level of *JAK3* + 0.028010873 × expression level of *PIK3CD* + 0.063966696 × expression level of *RPS6KA1* + 0.161467468 × expression level of *IKBKB* + 0.070073487 × expression level of *MDM2 +* 0.028624805 × expression level of *SOCS1*+ 0.251289822 × expression level of *NFKB1* + 0.241967045 × expression level of *ELK1* + 0.208932545 × expression level of *PCNA* + 0.046361918 × expression level of *IL2RG* + 0.308388697 × expression level of *IGFBP2* + 0.001870550 × expression level of *FES* + 0.009765477 × expression level of *HSP90B1*). The levels of gene expression for the 16 genes are comprehensively documented in Supplementary Table S1. The patients were then divided into low-risk and high-risk subgroups based on the median IGF1RS. The t-SNE results revealed a general alignment between the subgroups and a two-dimensional t-SNE distribution pattern (Figure 2C). Notably, the expression of the majority of genes exhibited a positive correlation with IGF1RS, except for *IGF1R*, which displayed low expression in the high-risk group (Figure 2D). Moreover, the low- and high-risk groups exhibited significant differences in the distribution of age (*p* < 0.001), tumor grade (*p* < 0.001), *IDH* status (*p* < 0.001), and 1p/19q status (*p* < 0.001) (Table 1). Moreover, the expression of the 16 genes exhibited significant differences in terms of clinicopathological characteristics (Supplementary Figure S2). Data from CGGA325 and CGGA693 cohorts were used to validate these findings (Supplementary Figures S3–S5, Supplementary Tables S2, S3).

**TABLE 1:** Characteristics of patients between high- and low-risk groups in TCGA-GBMLGG cohort.

Characteristics	N	High (N = 301)	Low (N = 302)	*p*-value
Grade	603			<0.001
WHO II		31(5.14%)	182(30.18%)	
WHO III		118(19.57%)	120(19.90%)	
WHO IV		152(25.21%)	0(0.00%)	
Gender	603			0.96
Female		126(20.90%)	128(21.23%)	
Male		175(29.02%)	174(28.86%)	
Age	603			<0.001
>41		232(38.47%)	125(20.73%)	
≤41		69(11.44%)	177(29.35%)	
IDH mutation status	597			<0.001
Mutant		78(13.07%)	295(49.41%)	
Wildtype		218(36.52%)	6(1.01%)	
1p/19q codeletion status	598			<0.001
Codeletion		16(2.68%)	133(22.24%)	
Non-codeletion		280(46.82%)	169(28.26%)	

### 3.2 Survival analysis and independent prognostic values of IGF1RS

**FIGURE 3:**
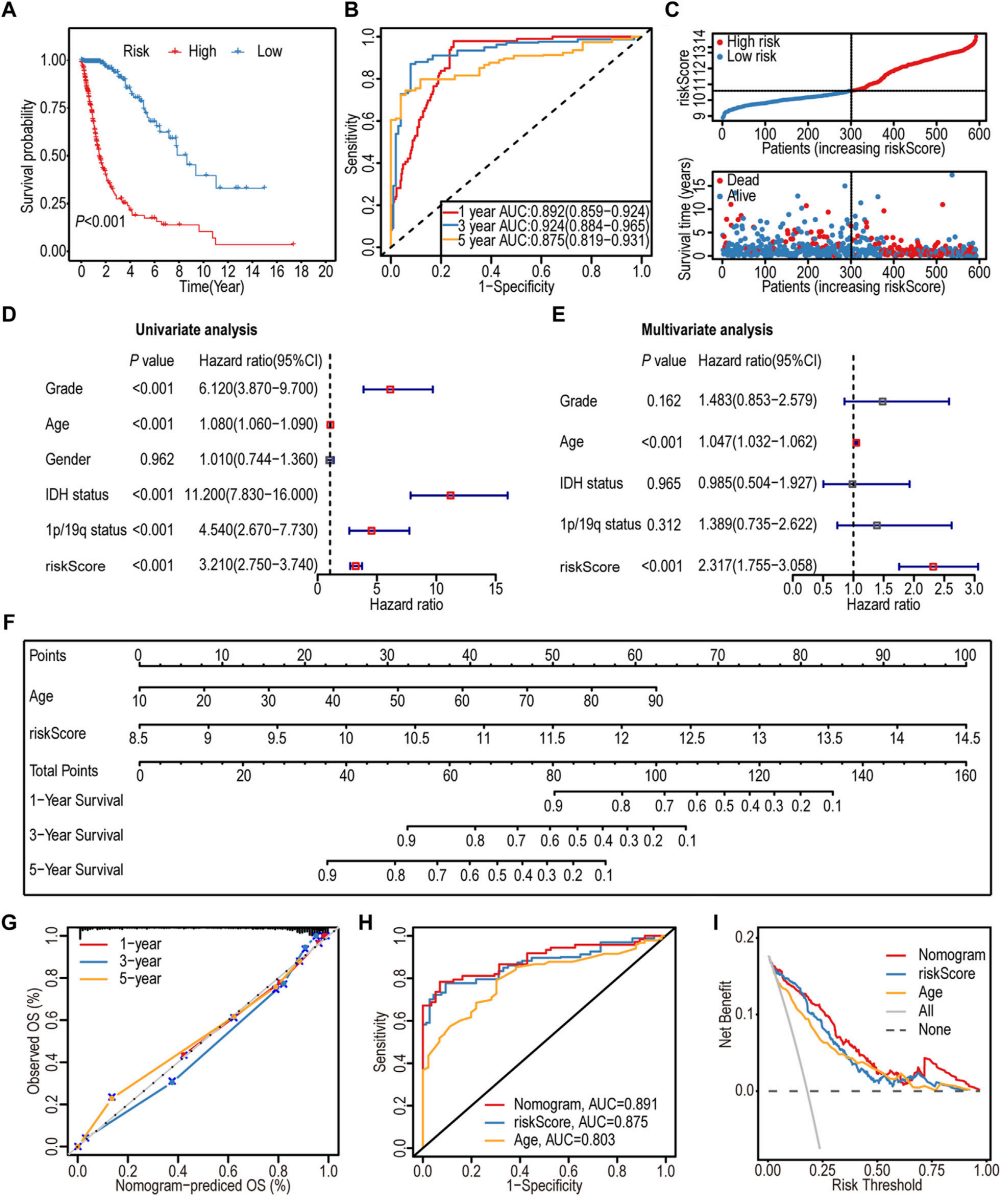
Prognostic significance of the 16-gene signature-derived risk score in the TCGA-GBMLGG cohort. **(A)** Kaplan-Meier analysis of the overall survival between low- and high-risk subgroups. **(B)** ROC curves to predict the sensitivity and specificity of 1-, 3-, and 5-year survival. **(C)** Ranked dot and scatter plots showing the distribution of risk score and patient survival status. The black dotted line is the optimal cut-off value for dividing patients into low- and high-risk groups. **(D)** Forest plot shows the associations between risk factors and survival of gliomas by univariate Cox regression analysis. **(E)** Forest plot shows that the risk signature is an independent predictor by multivariate Cox regression analysis. **(F)** A nomogram of the gliomas (TCGA-GBMLGG cohort) was used to predict the overall survival. **(G)** Calibration maps were used to predict the 1-, 3-, and 5-year survival. The *x*-axis and *y*-axis represent the predicted and actual survival rates of the nomogram, respectively. The solid line represents the predicted nomogram, and the vertical line represents the 95% confidence interval. **(H)** ROC curve analysis between nomogram and the significative characteristics (risk score and age) from multivariate Cox regression analysis. **(I)** DCA curves of the nomogram and significative characteristics (risk score and age). ROC: Receiver operating characteristic; DCA: Decision curve analysis.

The K–M survival analysis indicated a significant association between the high-IGF1RS group and poor outcomes (*p* < 0.001) (Figure 3A). Furthermore, IGF1RS exhibited favorable predictive capability for the 1-, 3-, and 5-year OS rates, with corresponding AUC values of 0.892, 0.924, and 0.875, respectively (Figure 3B). The ranked dot and scatter plots revealed that with an increase in the expression of IGF1RS, the survival time significantly decreases (Figure 3C). Similarly, IGF1RS was found to have a significant association with OS in the CGGA325 and CGGA693 cohorts (Supplementary Figure S6); these findings were consistent with those from the TCGA-GBMLGG cohort.

The association between IGF1RS and clinicopathological features was assessed using univariate and multivariate Cox regression analyses. As shown by the forest plots, similar to age, IGF1RS could serve as an independent prognostic factor (*p* < 0.001, HR: 2.317, 95% CI: 1.755–3.058) (Figures 3D, E).

### 3.3 Construction and evaluation of individualized prognostic prediction models

Recognizing the significance of both IGF1RS and age, a nomogram was developed to enhance the accuracy of individual prognosis prediction (C index: 0.874) (Figure 3F). The calibration curves for the nomogram at 1-, 3-, and 5-year intervals, as depicted in the plot (Figure 3G), demonstrated the accurate prediction of survival time for patients with glioma. Furthermore, we employed ROC analysis to assess the sensitivity of the prognostic prediction model. The AUC values for the nomogram, IGF1RS, and age at 5 years were found to be 0.891, 0.875, and 0.803, respectively (Figure 3H), indicating that the model was more effective in differentiating between patients with favorable or unfavorable prognoses than a single independent prognostic factor. The DCA curves further substantiated the superior predictive capability of the nomogram in prognosis assessment (Figure 3I).

### 3.4 Functional enrichment analysis related to IGF1RS

**FIGURE 4:**
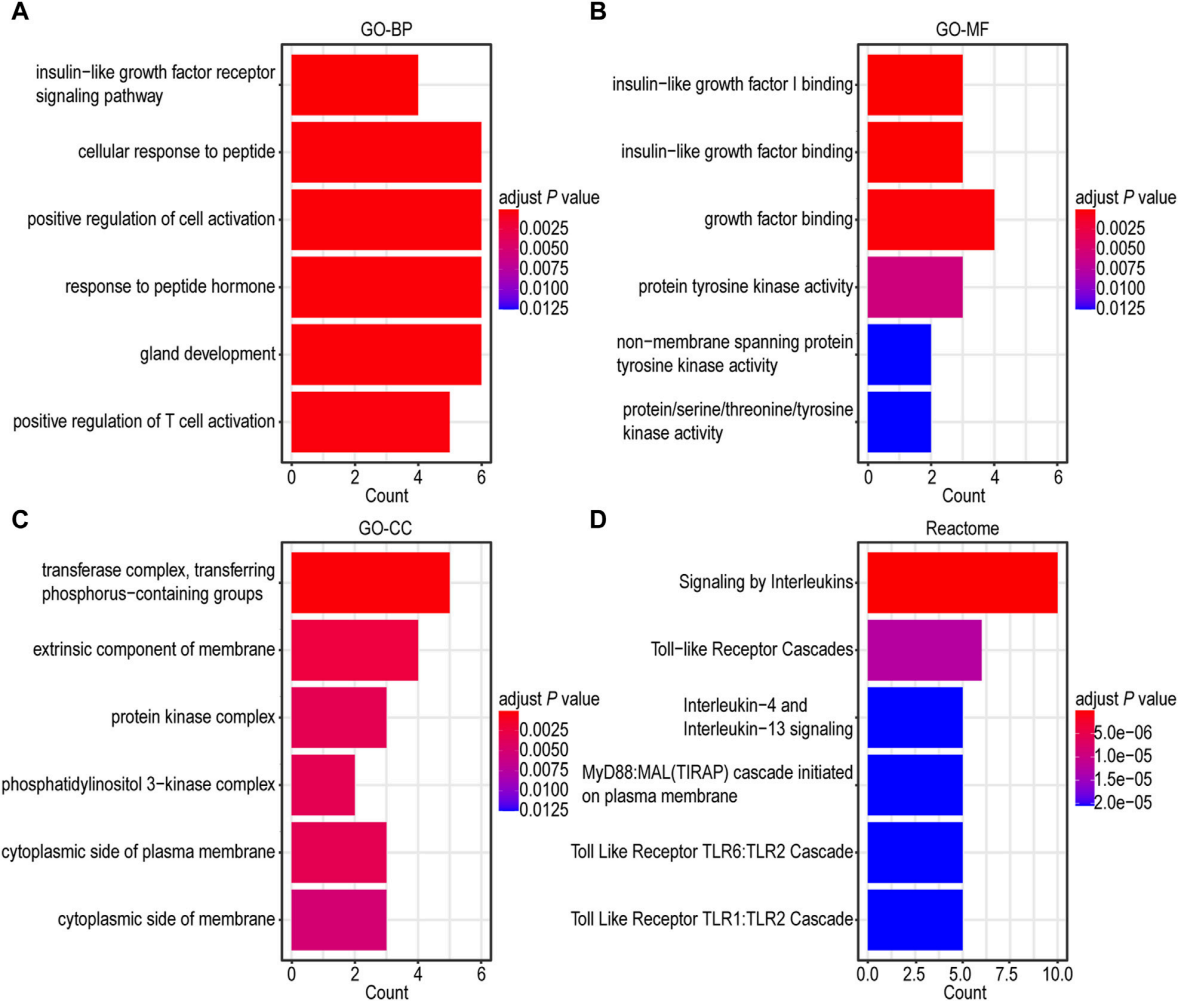
Functional enrichment analysis of genes related to risk score in TCGA-GBMLGG cohort. **(A)** Barplot graph for GO-BP. **(B)** Barplot graph for GO-MF. **(C)** Barplot graph for GO-CC. **(D)** Barplot graph for Reactome pathways.

To gain a more comprehensive understanding of the underlying biological functions and pathways associated with IGF1RS, we performed GO and Reactome pathway analyses using 16 risk genes. A Spearman’s correlation coefficient was employed for GO and Reactome pathway analyses. The gene ontology-biological processes (GO-BP) were mainly related to insulin-like growth factor receptor signaling pathway, cellular response to peptide, positive regulation of cell activation, response to peptide hormone, gland development, and positive regulation of T cell activation (Figure 4A). The enriched gene ontology-molecular functions (GO-MFs) were mainly related to insulin-like growth factor I binding, insulin-like growth factor binding, growth factor binding, protein tyrosine kinase activity, nonmembrane spanning protein tyrosine kinase activity, and protein/serine/threonine/tyrosine kinase activity (Figure 4B). The enriched gene ontology-cellular component (GO-CC) included the transferase complex, transfer of phosphorus-containing groups, extrinsic component of the membrane, protein kinase complex, phosphatidylinositol 3-kinase complex, cytoplasmic side of the plasma membrane, and cytoplasmic side of the membrane (Figure 4C). The Reactome pathway enrichment components were mainly related to signaling by interleukins, toll-like receptor cascades, interleukin-4 and interleukin-13 signaling, MyD88 and Mal/TIRAP cascade initiated on the plasma membrane, toll-like receptor TLR6/TLR2 cascade, and toll-like receptor TLR1/TLR2 cascade (Figure 4D). The genes associated with risk score were mainly involved in kinase itself, kinase-binding protein, and kinase regulation, which are consistent with the further function analysis of the genes comprising IGF1RS. Additionally, there is a close association between risk genes and immune-related pathways such as positive regulation of T cell activation (Baserga, 1995; Wu et al., 2011; Baxter, 2014). To further demonstrate the potential biological function of IGF1RS, we focused on immune microenvironment.

### 3.5 Correlation of IGF1RS with the immune microenvironment

**FIGURE 5:**
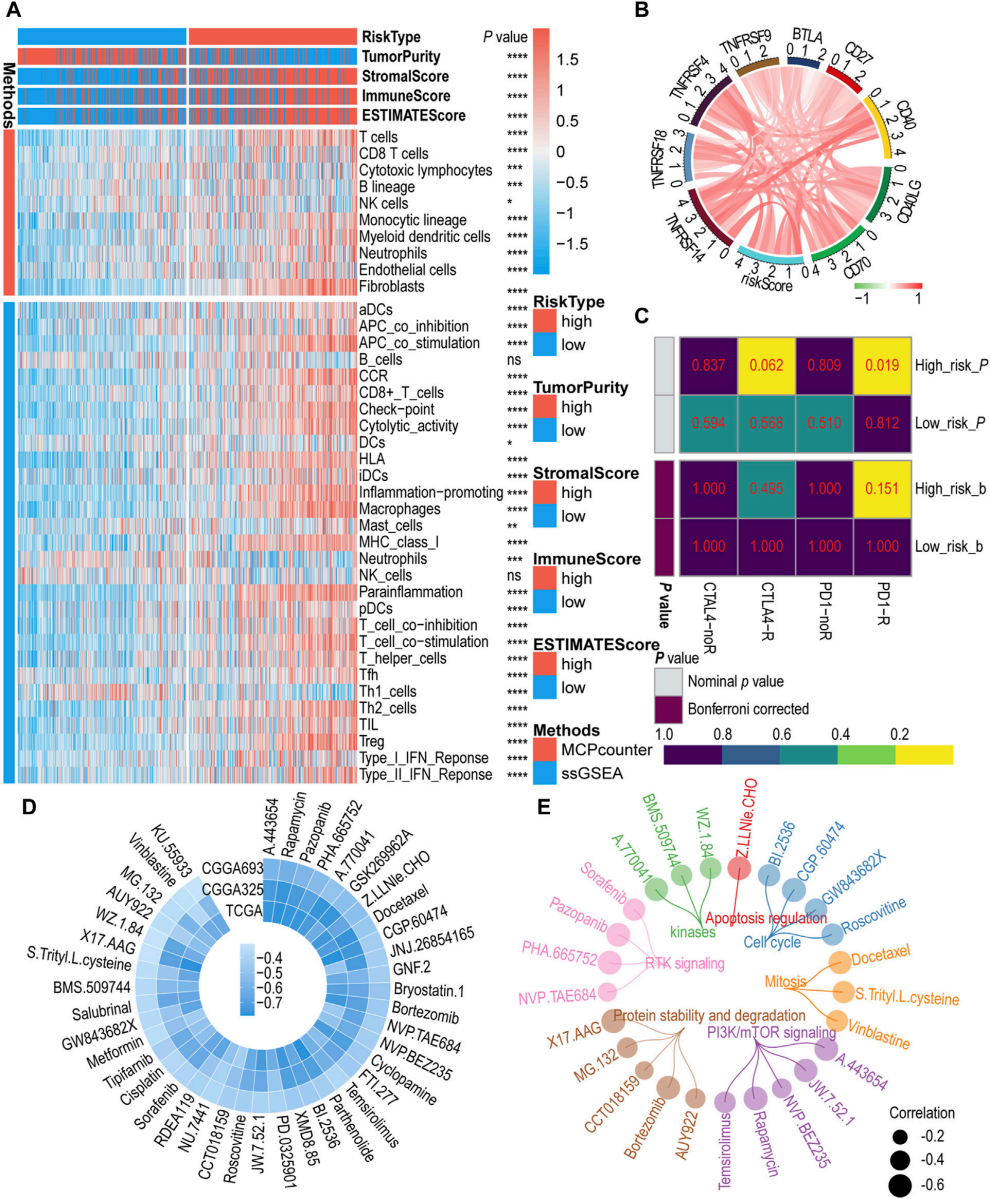
The role of risk signature in immune microenvironment, immunotherapy, and chemotherapy. **(A)** Immune cells infltration by MCP counter and ssGSEA algorithm between low- and high-risk subgroups in the TCGA-GBMLGG cohort. **(B)** Correlation between immune checkpoint genes and risk score in the TCGA-GBMLGG cohort. Red color indicates positive correlations, green color represents negative correlations, while white color is assigned to the median (correlation = 0). **(C)** Predicting response to immunotherapy (anti-PD1 and anti-CTLA4) in low- and high-risk subgroups based on the Submap algorithms in TCGA-GBMLGG cohort. The color of the grid in Submap heatmap represents the correlation *p*-value. **(D)** The circos plot illustrates the correlation coefficient (r > 0.3) calculated based on the IC50 and risk score in public cohorts. **(E)** The circos plot depicts a comprehensive representation of 25 drugs based on their target pathways. The *p-*value is indicated in the figure. ns: no significance; *: *p* < 0.05; **: *p* < 0 .01; ***: *p* < 0.001; ****: *p* < 0.0001.

To assess the relationship between IGF1RS and TIME, we investigated the correlation between IGF1RS and immune status, immune cell infltration, and immune cell subpopulations in the glioma microenvironment using the MCP and ssGSEA algorithms in the TCGA-GBMLGG (Figure 5A), CGGA325 (Supplementary Figure S7A), and CGGA693 (Supplementary Figure S7B) cohorts. We used the ESTIMATE algorithm to calculate tumor purity and immune score. The findings of the ESTIMATE algorithm demonstrated that patients with elevated IGF1RS exhibited a notably heightened tumor immune score. Endothelial cells and fibroblasts were significantly enriched in the high-risk subtypes based on the MCP algorithm. According to the ssGSEA algorithm, the high-risk subtypes exhibited a significant enrichment of immune cells, including aDCs, macrophages, pDCs, T helper cells, Th2 cells, and regulatory T cells (Tregs), whereas the low-risk groups demonstrated an abundance of Th1 cells. Moreover, a statistically significant difference was observed in immune-related biological processes or molecular functions between the low-risk and high-risk subgroups. These included antigen-presenting cell costimulation, chemokine receptor, checkpoint, cytolytic activity, human leukocyte antigen, major histocompatibility complex class I, para-inflammation, T cell co-inhibition, T cell costimulation, type I interferon (IFN) response, and type II IFN response. The endothelial cells can be classified into four types, namely, arteries, capillaries, veins, and lymphatic endothelial cells (Geldhof et al., 2022). Similarly, fibroblasts were categorized into three types: steady state-like (SSL), mechanoresponsive (MR), and immunomodulatory (IM) fibroblast cells (Foster et al., 2022; Jain et al., 2023). The findings revealed significant differences among SSL fibroblast cells, MR fibroblast cells, arteries endothelial cells, and lymphatic endothelial cells in TCGA-GBMLGG, CGGA325, and CGGA693 cohorts (Supplementary Figure S8). Additionally, the EPIC algorithm was used to validate the results regarding endothelial cells and fibroblasts (Supplementary Figure S8). Subsequently, the correlations between immune checkpoint-related genes and IGF1RS were calculated. In Figure 5B, the majority of immune checkpoint genes are colored red as they exhibit a positive correlation with the risk score. The result showed that the genes (including CD276, CD274, and PDCD1LG2) had functions on T-cell co-inhibition contributing to tumor cell evasion were enriched in high-risk groups and become important targets for blockade-based immunotherapy in cancer (Figure 5B, Supplementary Figure S9); (Lee et al., 2017; Yearley et al., 2017; Wang et al., 2019).

### 3.6 Prediction of immunotherapy and chemotherapy response in glioma

The above findings suggest that patients with high IGF1RS levels may be favorable candidates for immunotherapy. Then, the Submap algorithm was employed to further investigate the correlation between the IGF1RS signature and the efficacy of immunotherapy. Within this algorithm, we compared the expression profiles of two patient groups (high- and low-risk) with a publicly available dataset on immunotherapy. This dataset encompasses expression data from 47 melanoma patients who underwent treatment with PD-1 immune checkpoint inhibitors or CTLA-4 immune checkpoint inhibitors (Hoshida et al., 2007). The results demonstrated that the high-risk group exhibited a more pronounced response to anti-PD-1 treatment than the low-risk group, with the *p* values for the three cohorts being less than 0.05 (TCGA-GBMLGG: *p* = 0.019, Figure 5C; CGGA325: *p* = 0.019, Supplementary Figure 10A; CGGA693: *p* = 0.004, Supplementary Figure 10B). According to the Bonferroni-corrected *p* values (*p* = 0.032), more reliable prediction results were observed in the CGGA693 cohort. To further assess the differences in response to chemotherapy among patients in the high- and low-risk subgroups, 64 potential drugs were screened based on the GDSC database. Subsequently, the correlation coefficient between the IC50 of these potential drugs and IGF1RS was calculated for each sample in the CCGA325, CGGA693, and TCGA-GBMLGG cohorts, and we found that the correlation between these potential drugs and IGF1RS were negative, while no positive correlation was observed. The drugs with correlations less than 0.3 were excluded in order to show significant differences, while drugs with coefficients greater than 0.3 were retained (Figure 5D). Based on the target pathways of the drugs from GDSC website, the results showed 25 drugs (negative correlation with IGF1RS), including those involved in the cell cycle (BI.2536, CGP.60474, GW843682X, and roscovitine), mitosis (docetaxel, S-trityl-L-cysteine, and vinblastine), PI3K/mTOR signaling (A.443654, JW.7.52.1, NVP.BEZ235, rapamycin, and temsirolimus), protein stability and degradation (AUY922, bortezomib, CCT018159, MG.132, and X17.AAG), receptor tyrosine kinase signaling (NVP.TAE684, PHA.665752, pazopanib, and sorafenib), kinases (A.770041, BMS.509744, and WZ.1.84), and apoptosis regulation (Z.LLNle.CHO) (Figure 5E; Supplementary Figures 10C, D). The detailed correlation coefficients are shown in Supplementary Table S4. To validate the correlations between IGF1RS and drugs, we utilized the cMap website (https://clue.io/query) to ascertain the associations between IGF1RS and potential drugs correlated with IGF1RS in different cancer cell lines. The connectivity score indicates the association, with a higher negative correlation indicating the potential to reverse the molecular characteristics of the disease and, theoretically, an increased likelihood of effectively treating the disease. The antitumor effects of 17 potential drugs were fully validated, and their high negative connectivity scores are shown in Supplementary Table S5.

### 3.7 The expressions of HSP90B1 in glioma

**FIGURE 6:**
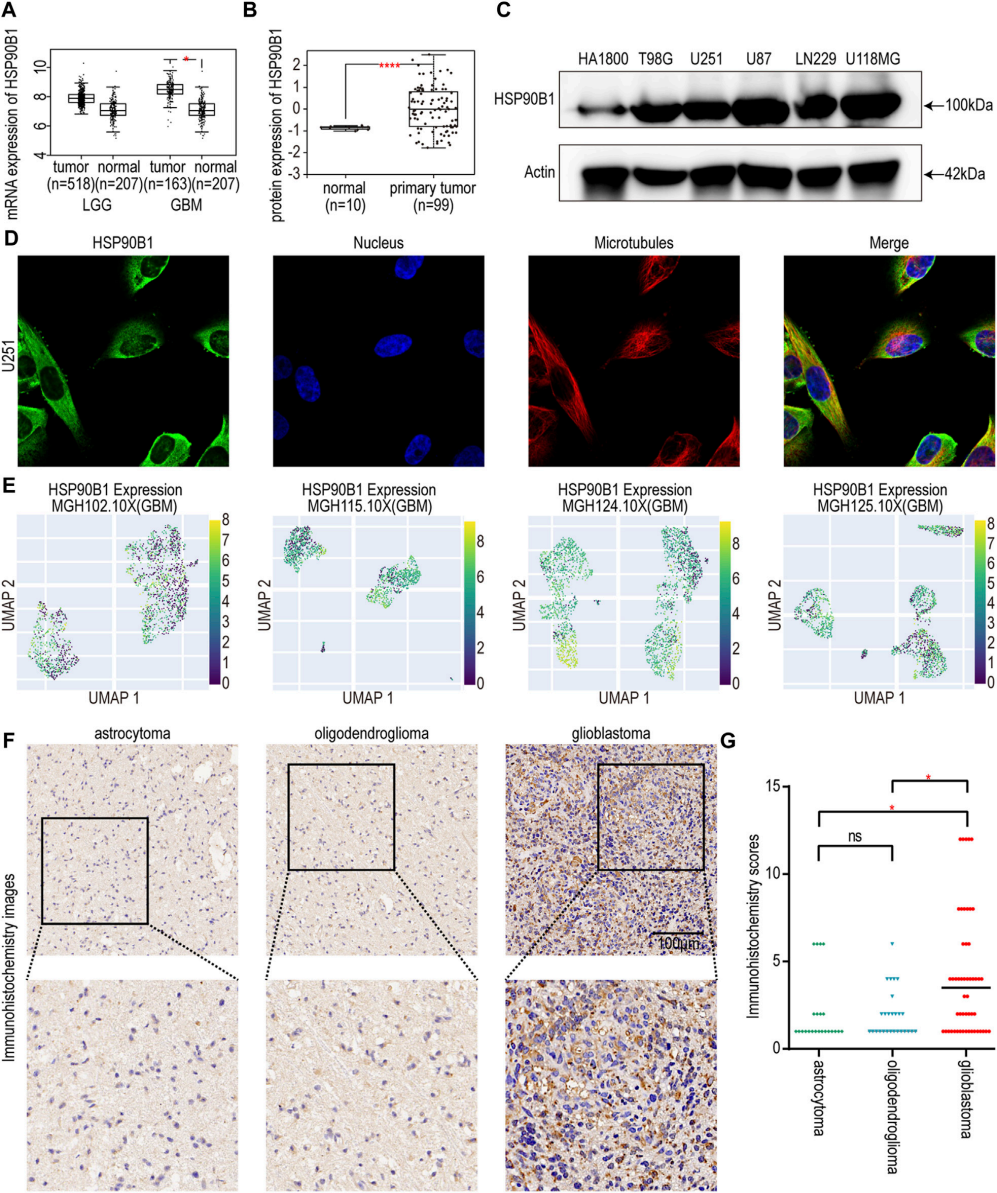
**(A)** Differences in *HSP90B1* mRNA expression in normal and cancerous tissues in LGG and GBM. **(B)** Differences in the expression of HSP90B1 protein in normal and primary gliomas. **(C)** Protein expression of HSP90B1 in HA 1800, T98G, U251, U87, LN229, and U118MG cell lines. **(D)** The immunofluorescence images depicting the HSP90B1 distribution in the U251 cell line from the HPA database. **(E)** UMAP plots of single-cell RNA-seq level expression of HSP90B1 in MGH102.10X, MGH115.10X, MGH124.10X, and MGH125.10X from the CHARTS database. **(F)** Representative immunohistochemistry images depicting HSP90B1 expression in astrocytoma, oligodendroglioma, and glioblastoma were obtained from a cohort of 101 glioma tissues collected at the First Affiliated Hospital of Hainan Medical University. The scale bar is 100 µm. **(G)** The Kruskal–Wallis test was used to determine if the IHC staining score of one group in in astrocytoma (n = 23), oligodendroglioma (n = 27), and glioblastoma (n = 51) had different distributions from the others respectively. LGG: low-grade gliomas; GBM: glioblastoma. The *p-*value is indicated in the figure. ns: no significance; *: *p* < 0.05; ****: *p* < 0.0001.

To screen and validate the expression of risk score-related in glioma, we performed literature investigation, assessed protein expression, and conducted druggability analysis. Among them, few relevant studies have been conducted on HSP90B1 in glioma. The HSP90B1 protein exhibits elevated expression levels in glioma according to the HPA database (Uhlén et al., 2015), indicating its potential role in the pathogenesis of this neurological malignancy. Subsequently, we conducted an analysis of HSP90B1 associated with drug target tractability using the DepMap website (https://depmap.org/portal/) and showed that the HSP90B1 protein possesses bioactive compounds and has a druggable structure. This heightened expression may suggest a significant association between the druggable protein HSP90B1 and glioma development, highlighting its importance as a potential therapeutic target for further investigation in glioma treatment strategies. Hence, HSP90B1 was chosen for further comprehensive investigation. The results of GEPIA database showed that the expression of *HSP90B1* in GBM tumor tissue was significantly higher than that in normal tissue, but there was no significant difference in LGG (Figure 6A). UALCAN database analysis revealed higher levels of HSP90B1 protein in GBM tissue compared to normal tissue (Figure 6B). To verify the protein expression level of HSP90B1, a Western blot assay was performed. Compared with the normal astrocyte cell line HA 1800, the protein expression levels of T98G, U251, U87, LN229, and U118MG were higher (Figure 6C). The HPA database was used to confirm the cytoplasmic localization of HSP90B1 in U251 human glioma cell lines by immunofluorescence (Figure 6D). Additional investigations were conducted to explore the expression of *HSP90B1* at the single-cell level, revealing predominant expression in myeloid cells and astrocytes (Figure 6E). Meanwhile, *HSP90B1* was found to be significantly enriched in subgroups characterized by low malignancy scores, and these particular subgroups exhibited higher inflammation scores (Zhuo et al., 2023). In addition, the immunohistochemical results revealed that the staining intensity of HSP90B1 protein in patients with GBM was more pronounced than that in patients with astrocytoma or oligodendroglioma (Figures 6F, G).

## 4 Discussion

IGF-1R, a molecule known to exert potential carcinogenic effects in various tumors, plays a crucial role in the IGF-1R signaling pathway (Chen et al., 2020; Hua et al., 2020). In the development of malignant tumors in humans, the signaling level of the IGF/IGF-1R pathway plays a pivotal role in promoting cell transformation, tumor cell proliferation, resistance to apoptosis, and metastasis (Scagliotti and Novello, 2012; Liefers-Visser et al., 2017). Considering the significant role played by IGF-1R signaling in carcinogenesis pathways (Chen et al., 2020; Hua et al., 2020) as well as the poor prognosis associated with glioma (Simpson et al., 2020), further investigation of the IGF-1R-related signaling pathway genes in glioma is necessary.

In this study, we used the *IGF-1R*-related signaling pathway genes to develop a predictive risk model for further investigation of their role in glioma. Our comprehensive analysis revealed an association between IGF1RS and immune infltration, and drug therapy. We found that IGF1RS could serve as an independent prognostic indicator for gliomas. Moreover, the development of a personalized prognostic model based on IGF1RS and age showed robust predictive performance, indicating significant potential in predicting the 5-year survival rate (AUC = 0.891). The IGF1RS signatures including 16 genes. Among them, *IGFBP1* and *IGFBP5* play important roles in transcriptional regulation, apoptosis induction and DNA damage repair, and can modulate *IGF-1R* signaling pathway activity upon binding to *IGF1/2* ligand (Baxter, 2014). Meanwhile, the *IGF-1R* signaling can be enhanced by non-receptor tyrosine adhesion kinase *FES*-related (FER) to facilitate cooperative growth and adhesion signaling that potentially contributing to cancer progression (Stanicka et al., 2018). The *IGF-1*/*IGF-1R* signaling can activate the PI3K/AKT/mTOR pathway signaling pathway (Abdel-Wahab et al., 2018), which is closely related to *AKT1* and *PIK3CD* and plays a critical role in glioma progression, promoting cancer cell proliferation and inducing drug resistance. In addition, the negative feedback modulating effect of *SOCS1* on *IGF-1R* mediated signaling has been reported (Inaba et al., 2005) and whether *SOCS1* can negatively inhibit *IGF-1R* signaling in gliomas is worthy of further study. The E3 ubiquitin ligase *MDM2* is believed to mediate the degradation of *IGF-1R*, thereby directly regulating its expression levels (Girnita et al., 2007). Conversely, *PCNA* indirectly modulates the expression level of *IGF-1R* by activating *PDK1* (Wu et al., 2011). In order to further elucidate the potential biological functions associated with IGF1RS, we conducted functional pathway analysis utilizing IGF1RS as opposed to individual genes. The results revealed a close association between risk genes and immune-related pathways, such as positive regulation of T cell activation. Glioma is renowned for its intricate TIME and poor response to immune checkpoint inhibitors, with T cell dysfunction favoring tumor immune evasion among patients with glioma (Mirzaei et al., 2017; Woroniecka et al., 2018). Consequently, we investigated the correlation between IGF1RS and immune scores along with immune cells and observed significant disparities in immune cell abundance between the high-risk and low-risk subgroups. Notably, immunosuppressive cells, such as Th2 cells and Tregs, were significantly enriched within the high-risk subgroup, whereas Th1 cells predominantly populated the low-risk subgroup. Furthermore, a positive correlation was identified between IGF1RS and TNF superfamily immune checkpoint-related genes as well as most B7-CD28 family genes. These findings suggest that cancer cells in patients with high-risk glioma may evade elimination by overexpressing immunosuppressants after stimulating immune activation. Overall, these findings suggest that IGF1RS plays a pivotal role in the TIME of gliomas and that immunotherapy can confer greater benefits on high-risk patients.

Next, we investigated the differences in the responses to immunotherapy between the high- and low-risk groups. Previous studies have demonstrated that patients with glioma exhibit limited sensitivity to immunotherapy (Yu and Quail, 2021). However, the identification of advanced immunotherapy regimens (Luo et al., 2023; Morimoto et al., 2023), and emerging treatment targets (Zhuo et al., 2023) is anticipated to overcome this obstacle. Upon examining the three cohorts, we observed that patients with an elevated IGF1RS exhibited a heightened response to anti-PD1 treatment. This implies that high-risk subtype patients may demonstrate enhanced sensitivity to immunotherapy. Subsequently, we used the predictive model of GDSC to assess chemotherapy drugs and analyze differences in response between the high- and low-risk subgroups. Interestingly, we discovered a negative correlation between IC50 values and IGF1RS, indicating the potential benefits of chemotherapy for high-risk patients. IGF-1R is a multifunctional membrane-associated tyrosine kinase (DeCordova et al., 2020), which potentially facilitates the glioma patient’s response to kinase-inhibiting drugs such as WZ.1.184, BMS.509744, and A770041. As for drugs involved in the PIK/mTOR signaling pathway (A.4JW.7.52.1, NVP.BEZ235, rapamycin, and temsirolimus), Quail et al. demonstrated that the activation of the PI3K pathway in recurrent GBM can be enhanced by both macrophage-derived IGF-1 and tumor cell IGF-1R (Quail et al., 2016), thereby indirectly confirming their potential anticancer effects within the context of the IGF-1R signaling pathway. This undoubtedly provides a new chemotherapy pathway for gliomas.

Among the genes involved in establishing IGF1RS, we observed that the exploration of *HSP90B1* in gliomas was relatively limited. HSP90B1, a member of the heat shock protein (HSP) 90 family (Chen et al., 2005), is a stress-induced molecular chaperone that facilitates the normal folding, intracellular disposition, and proteolytic turnover of numerous key regulators involved in cell growth, differentiation, and survival (Whitesell and Lindquist, 2005). It plays a crucial role in IGF production and signaling (Radosevic-Stasic et al., 2012) and Wnt pathway (Liu et al., 2013). During oncogenesis, tumor cells exhibit an increased reliance on HSPs (including HSP90B1) for chaperoning to support their proliferation due to the misfolding of oncoproteins requiring enhanced chaperone activity for correct folding (Chatterjee and Burns, 2017). This phenomenon has been confirmed by elevated expression levels of *HSP90B1* observed in various cancer tissues (Dejeans et al., 2012; Rachidi et al., 2015). We found significant differences in the expression of *HSP90B1* between GBM and normal tissue. Using staining analysis of tissue from 101 glioma patients, the HSP90B1 protein was found to be significantly higher in tumor sections in GBM patients than in astrocytoma or oligodendrocytoma patients. This indicates its important role in glioma progression. The inhibition of HSP90B1 in targeted therapy may impede the correction of misfolded oncoproteins, thereby inducing cancer cell death. And various inhibitors have been developed as anticancer agents (Whitesell and Lindquist, 2005; Taipale et al., 2010; Patel et al., 2013). For instance, DN401 exhibits robust inhibition of HSP90B1 both *in vitro* or *in vivo* (Park et al., 2020). After conducting predictive analysis on an extensive range of drugs utilizing the GDSC database, we have successfully identified 25 distinct drugs based on the target pathways, thereby establishing a robust foundation for targeted drug exploration of HSP90B1.

Despite these findings, this study had some limitations. For example, the prognostic value of IGF1RS requires further validation in clinical cohorts. In addition, the role and function of *HSP90B1* as a prognostic biomarker in glioma should be further investigated using 3D cell culture and cell- or patient-derived xenograft models.

## Data Availability

The dataset presented in this study can be found in online repositories. The names of the repository/repositories and accession number(s) can be found in the article/Supplementary Material.
