# Supplementary material for: Prognosis and therapeutic significance of IGF-1R-related signaling pathway gene signature in glioma
Source: Front Cell Dev Biol. 2024 Apr 11;12:1375030. doi: 10.3389/fcell.2024.1375030 (PMC11043541; doi:10.3389/fcell.2024.1375030)
Supplement: Supplementary file 2 [file DataSheet1.docx]

**
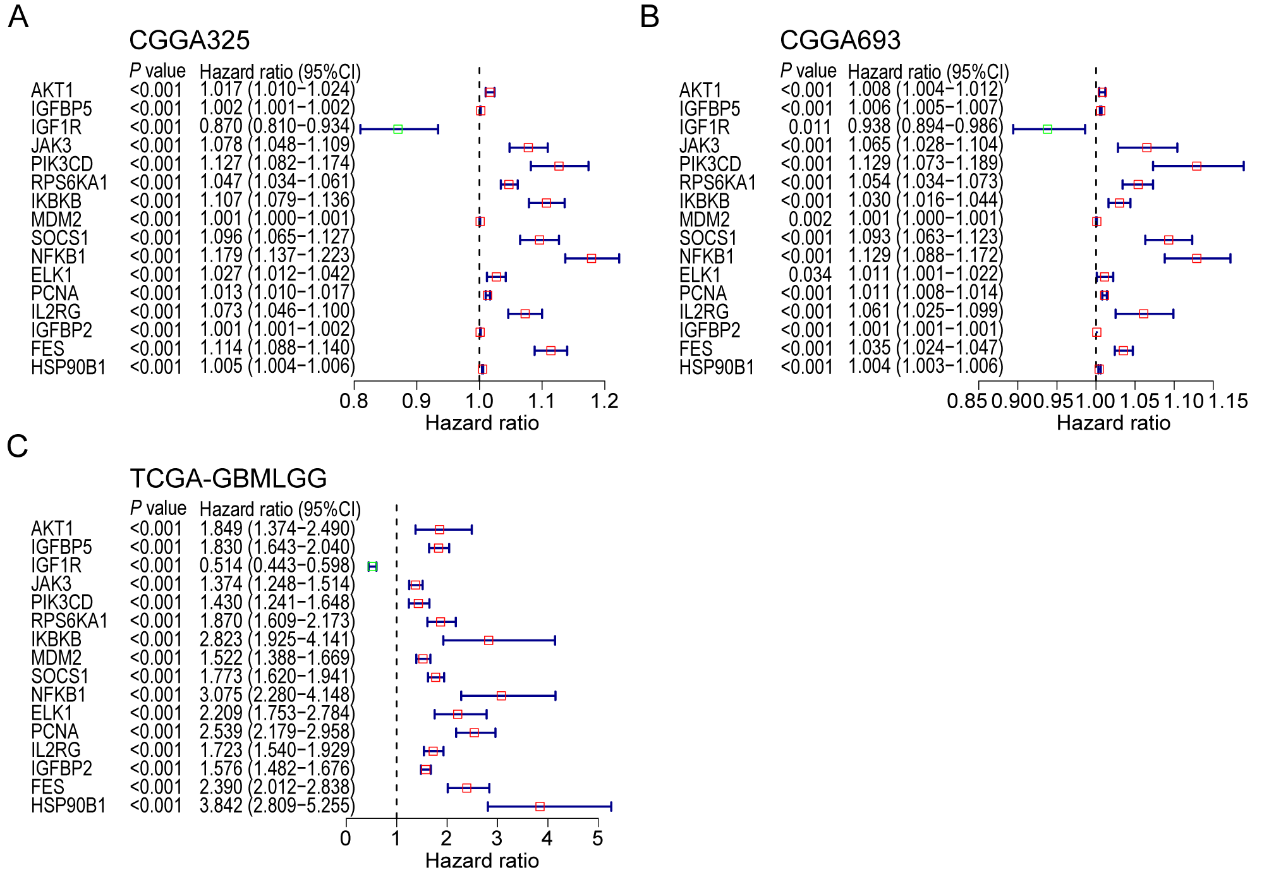
 Prognosis and** **Therapeutic Significance of IGF-1R-related** **Signaling Pathway Gene Signature in Glioma**

**Supplementary Figure S1** The forest plots of 16 genes for univariate Cox regression analyses from CGGA325 (**A**), CGGA693 (**B**), and TCGA-GBMLGG (**C**) cohorts. 95% CI: 95% confidence intervals.

**
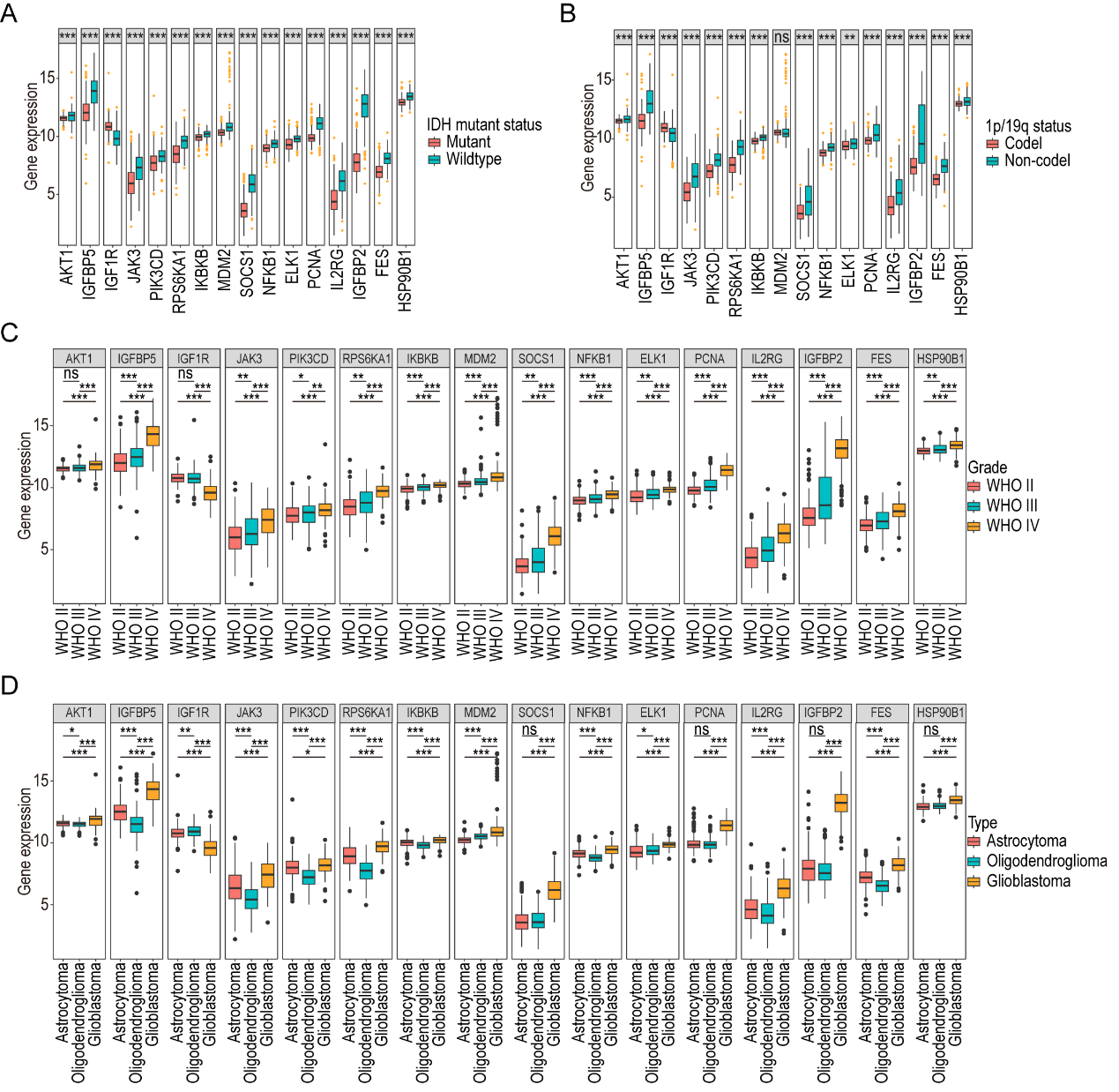
**

**Supplementary Figure S2** The difference in the expression of 16 genes in different clinicopathological characteristics (IDH mutant status **(A)**; 1p/19q status **(B)**; Grade **(C)**; Type **(D)**) based on the TCGA-GBMLGG cohort. ns: no significance; *: *P* < 0.05; **: *P* < 0 .01; ***: *P* < 0.001.


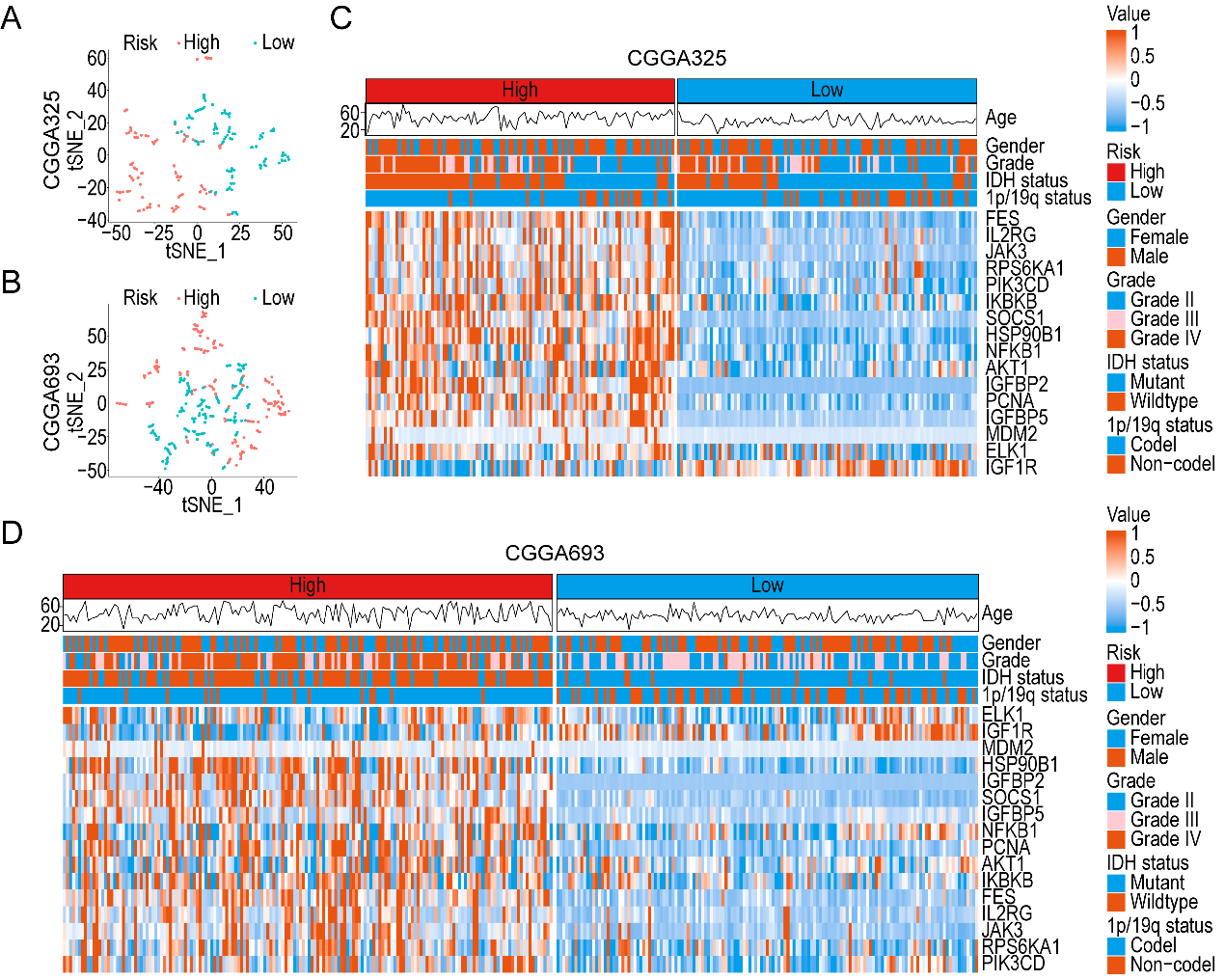
**Supplementary Figure S3** The t-SNE analysis supported the stratification into low-risk and high-risk subgroups based on the CGGA325 **(A)** and CGGA693 **(B)** cohorts. Heatmap shows the association between risk and clinicopathological features based on the 16-gene risk signature in the CGGA325 **(C)** and CGGA693 **(D)** cohorts.


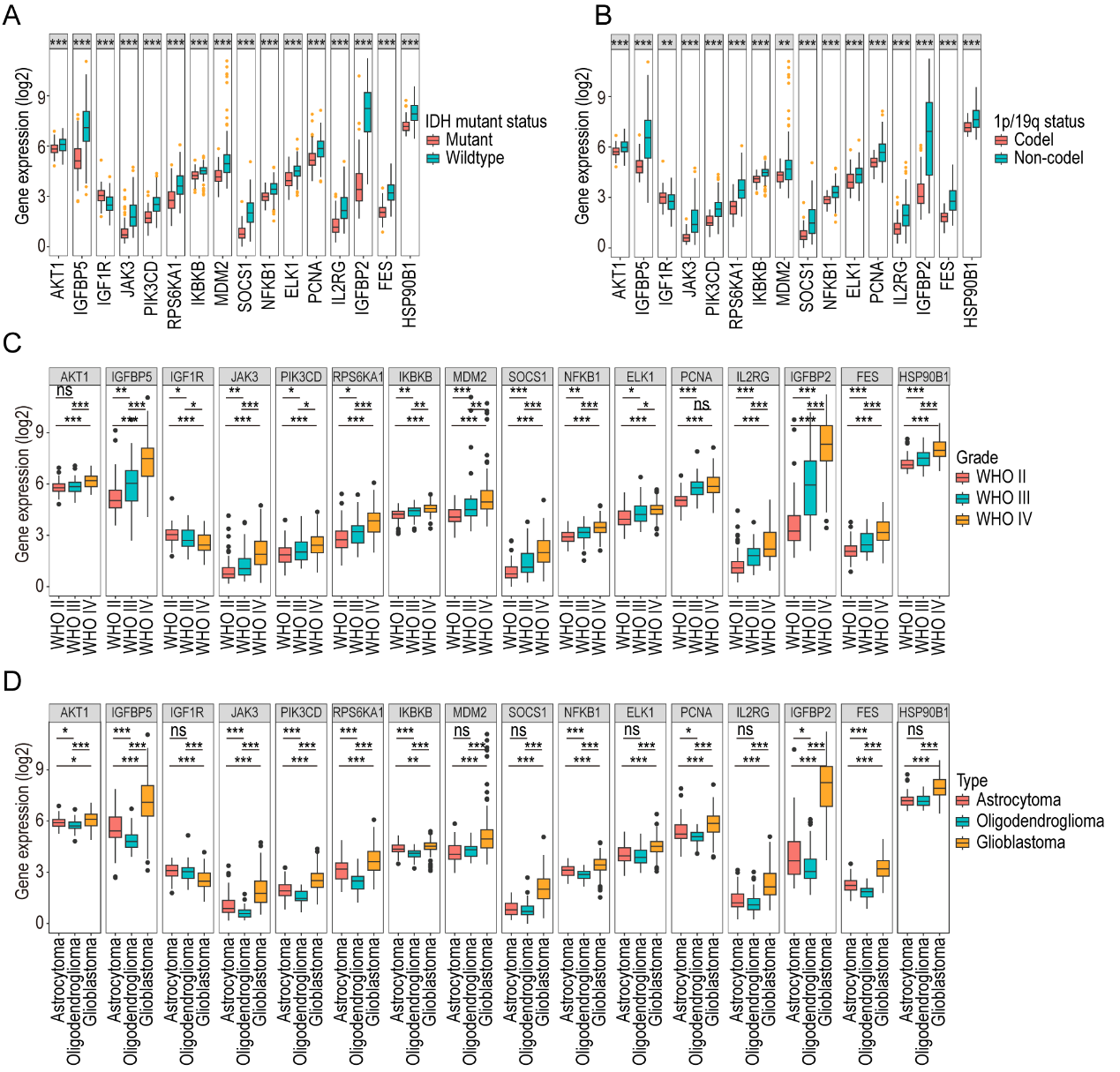
**Supplementary Figure S4** The difference in the expression of 16 genes in different clinicopathological characteristics (IDH mutant status **(A)**; 1p/19q status **(B)**; Grade **(C)**; Type **(D)**) based on the CGGA325 cohort. ns: no significance; *: *P* < 0.05; **: *P* < 0 .01; ***: *P* < 0.001.


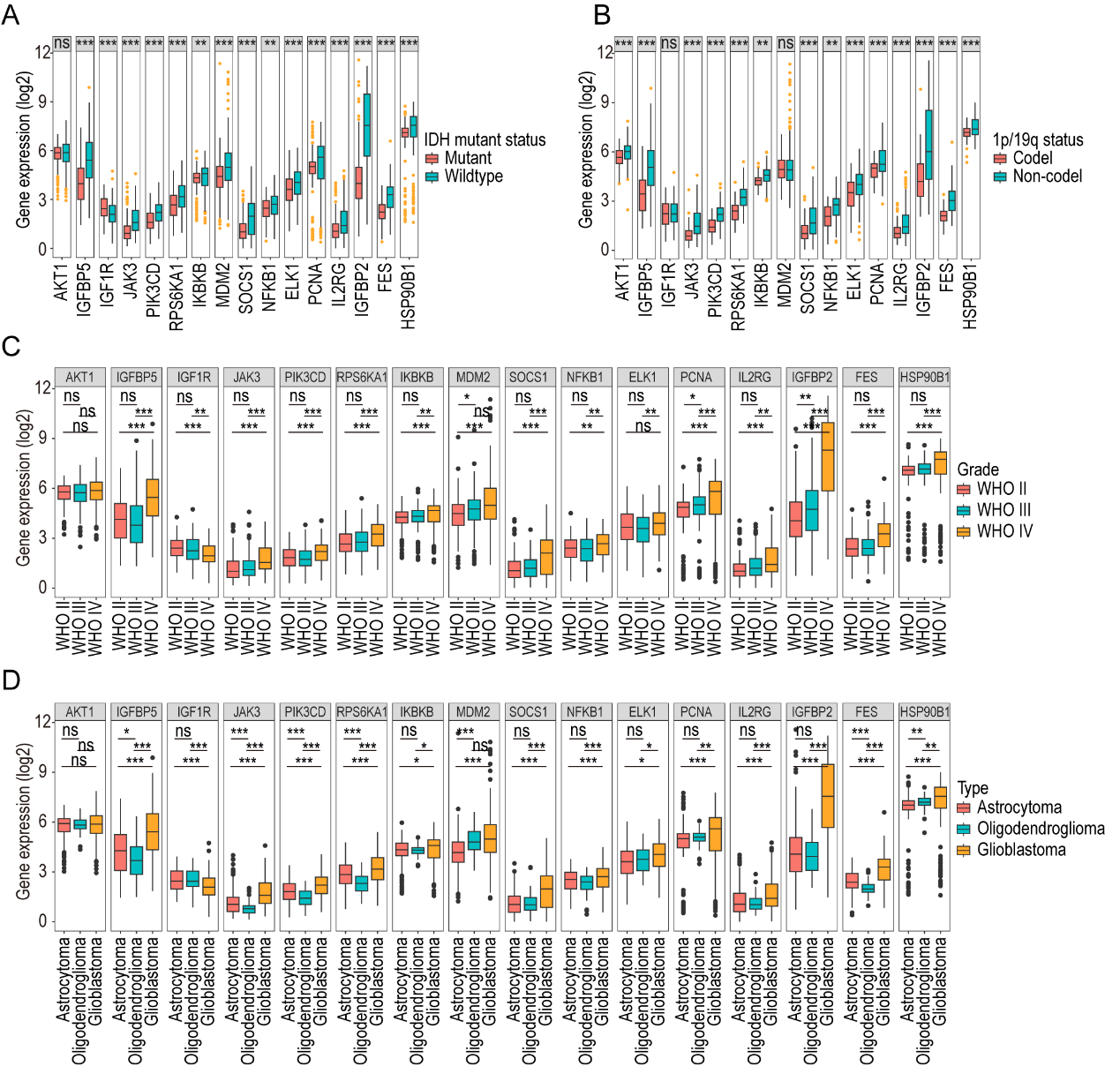
**Supplementary Figure S5** The difference in the expression of 16 genes in different clinicopathological characteristics (IDH mutant status **(A)**; 1p/19q status **(B)**; Grade **(C)**; Type **(D)**) based on the CGGA693 cohort. ns: no significance; *: *P* < 0.05; **: *P* < 0 .01; ***: *P* < 0.001.


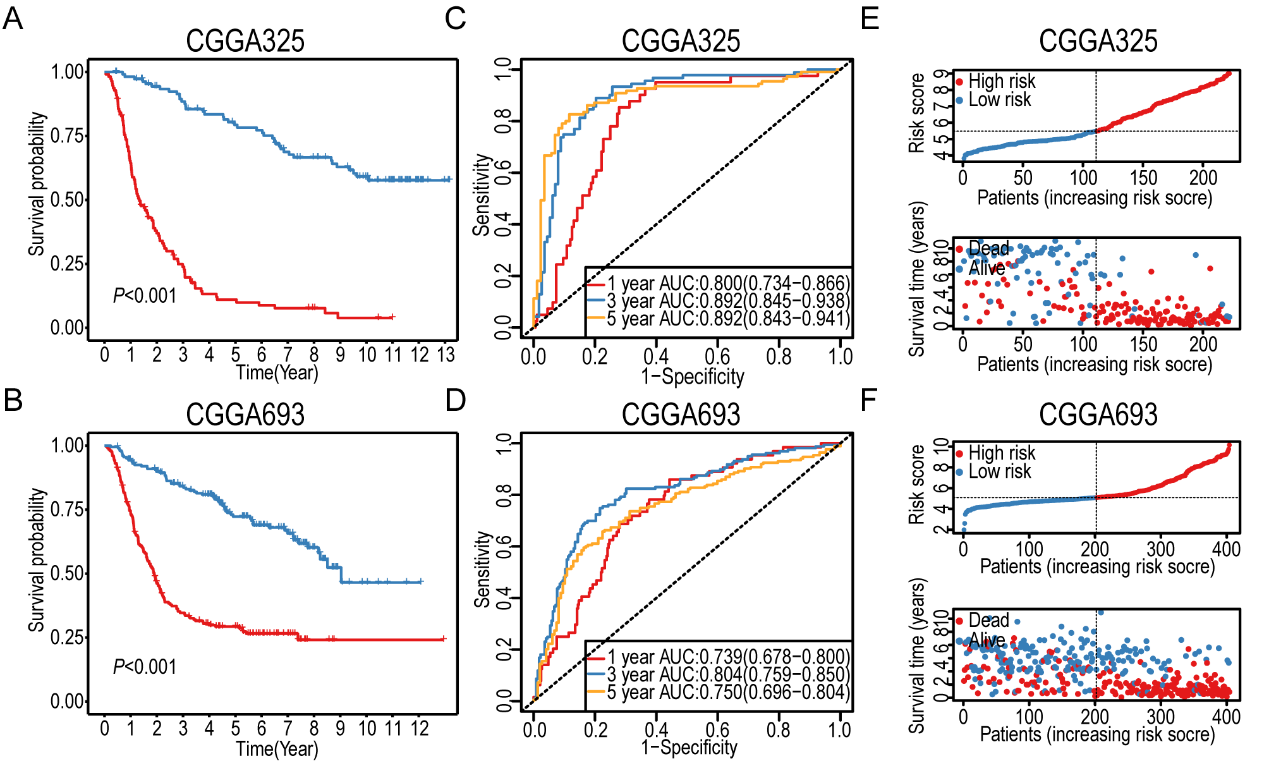
**Supplementary Figure S6** Kaplan-Meier analysis of the overall survival between low- and high-risk subgroups based on the CGGA325 **(A)** and CGGA693 **(B)** cohorts. ROC curves to predict the sensitivity and specificity of 1-, 3-, and 5-year survival based on the CGGA325 **(C)** and CGGA693 **(D)** cohorts. Ranked dot and scatter plots showing the distribution of risk score and patient survival status based on the CGGA325 **(E)** and CGGA693 **(F)** cohorts. The black dotted line is the optimal cut-off value for dividing patients into low- and high-risk groups.

**Supplementary Figure S7** Immune cells infiltration by MCP counter and ssGSEA algorithm between low- and high-risk subgroups in the CGGA325 **(A)** and CGGA693 **(B)** cohorts. ns: no significance; *: *P* < 0.05; **: *P* < 0 .01; ***: *P* < 0.001; ****: *P* < 0.0001
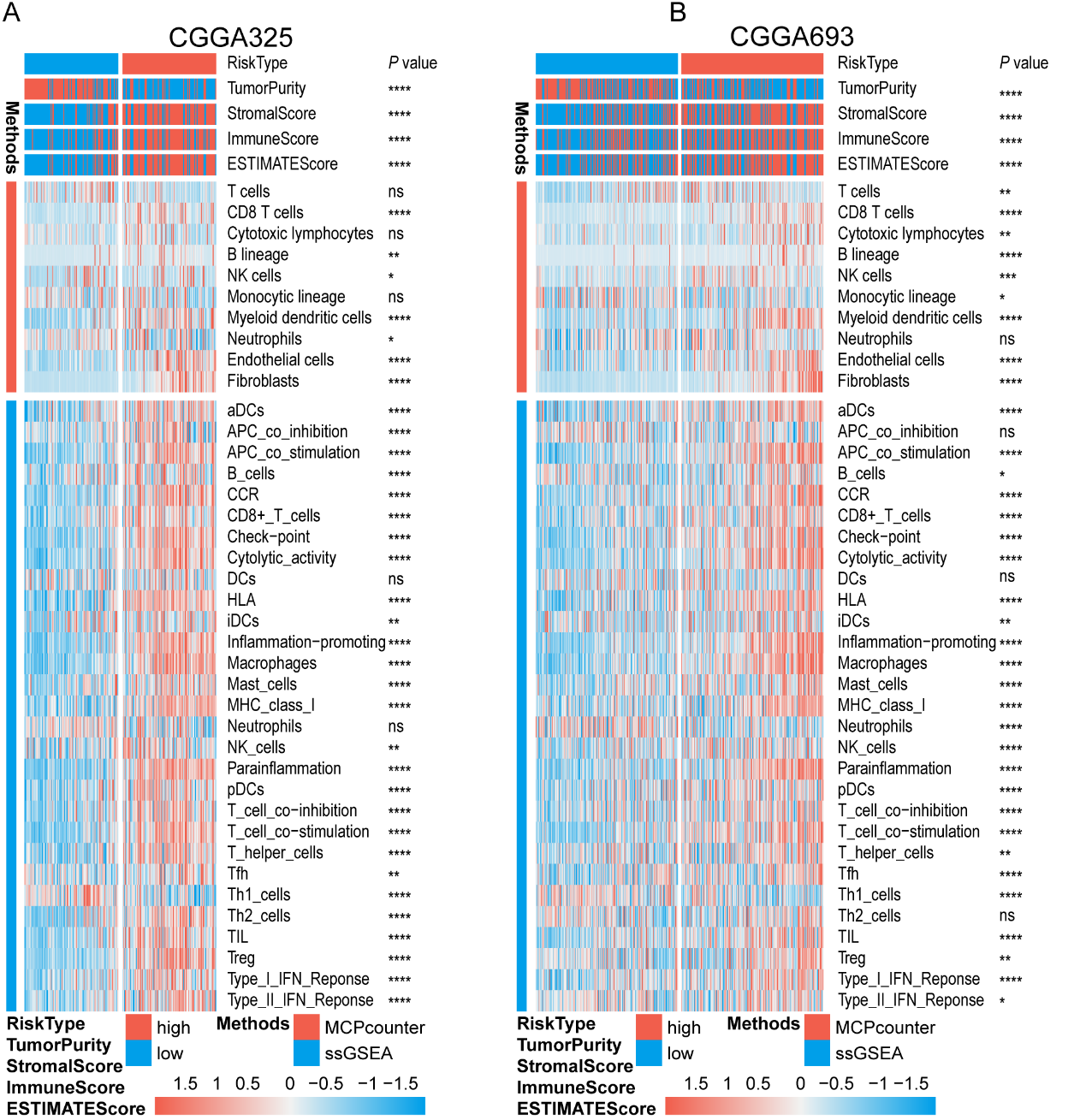
.


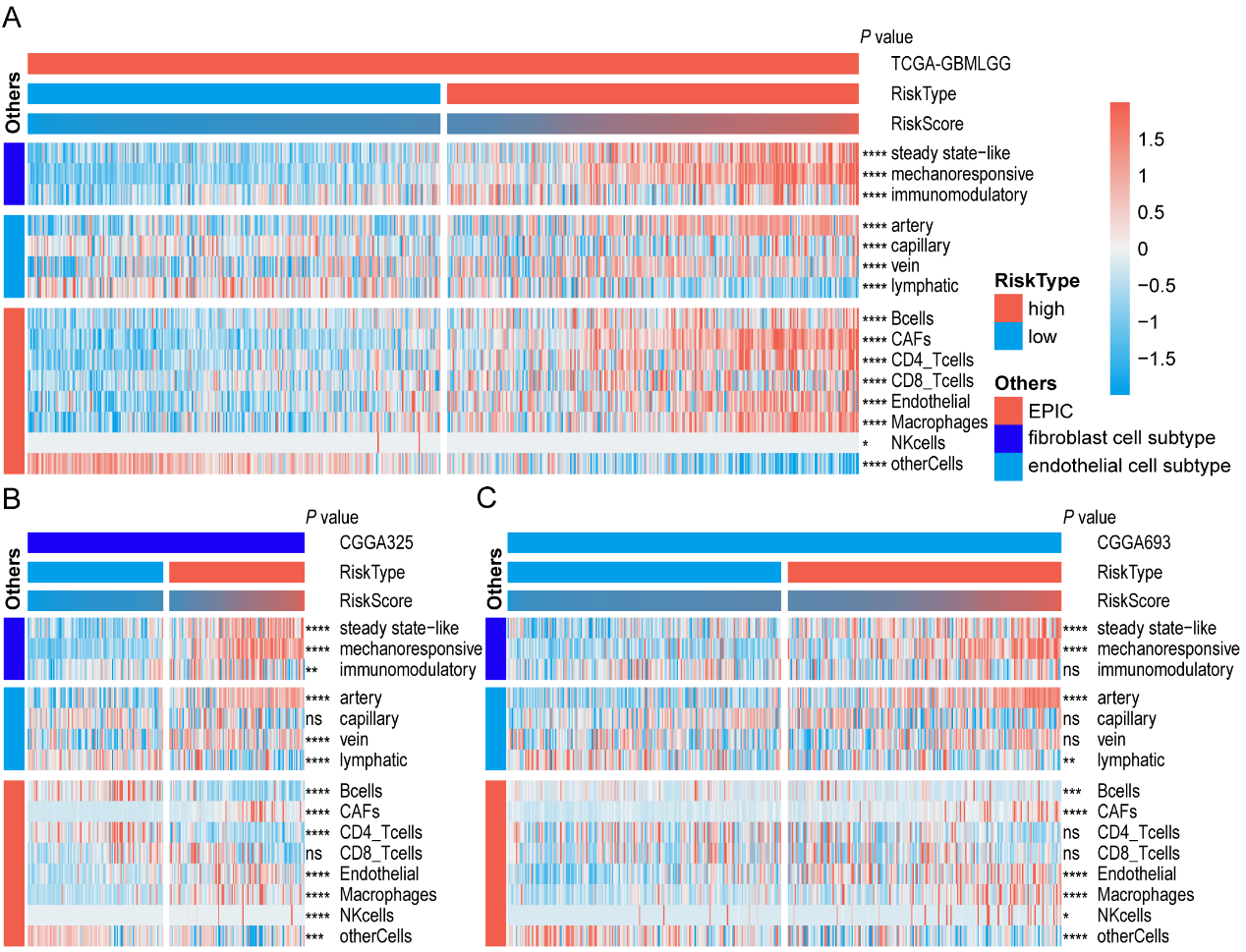


**Supplementary Figure S8** The heapmap of fibroblast cell subtype, endothelial cell subtype, and immune cells infiltration by EPIC algorithm in the TCGA-GMBLGG **(A)**, CGGA325 **(B)**, and CGGA693 **(C)** cohorts. ns: no significance; *: *P* < 0.05; **: *P* < 0 .01; ***: *P* < 0.001; ****: *P* < 0.0001.


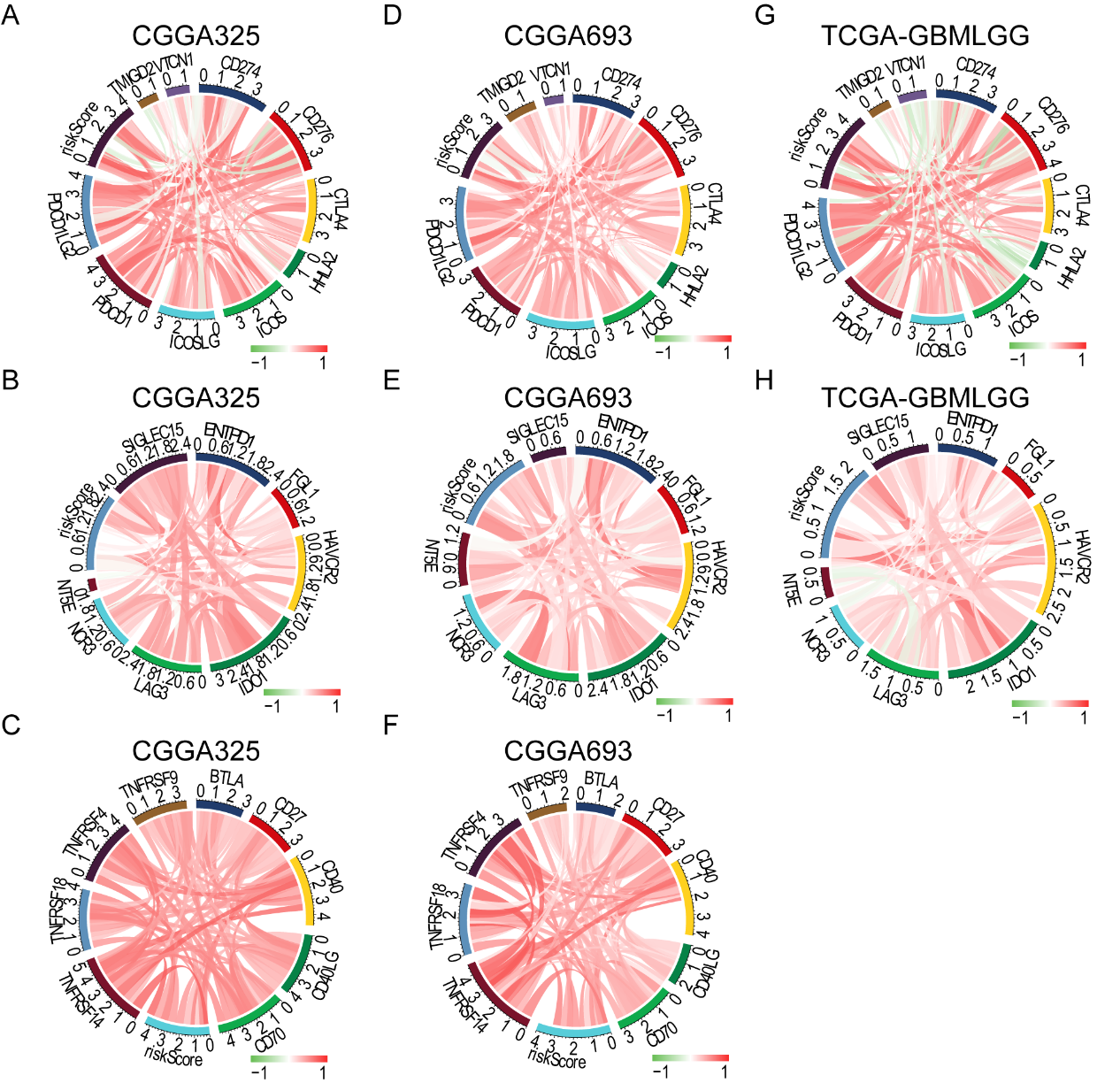
**Supplementary Figure S9** Correlation between immune checkpoint genes and risk score in the CGGA325 **(A-C)** and CGGA693 **(D-F)**, and TCGA-GBMLGG **(G-H)** cohorts. Red color indicates positive correlations, green color represents negative correlations, while white color is assigned to the median (correlation = 0).


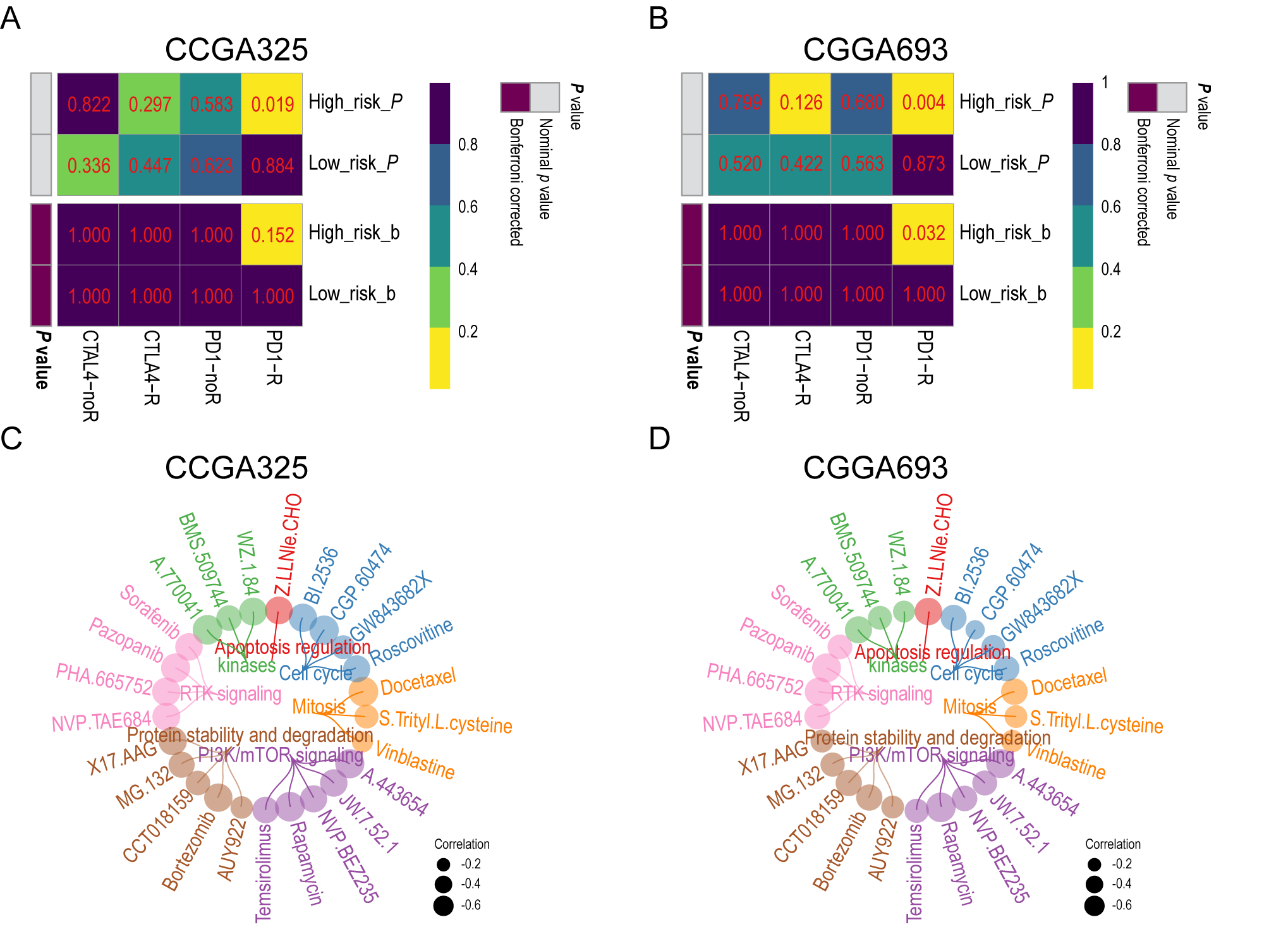
**Supplementary Figure S10** Predicting response to immunotherapy (anti-PD1 and anti-CTLA4) in low- and high-risk subgroups based on the Submap algorithms in the CGGA325 **(A)** and CGGA693 **(B)** cohorts. The circos plots depict a comprehensive representation of 25 drugs based on their target pathways in the CGGA325 **(C)** and CGGA693 **(D)** cohorts.

**Supplementary Table S2** Characteristics of patients between high- and low-risk groups in CGGA325 cohort.

| Characteristics | N | high (N=111) | low (N=111) | *P* value |
| --- | --- | --- | --- | --- |
| Grade | 222 |  |  | <0.001 |
| WHO II |  | 6(2.70%) | 84(37.84%) |  |
| WHO III |  | 29(13.06%) | 18(8.11%) |  |
| WHO IV |  | 76(34.23%) | 9(4.05%) |  |
| Gender | 222 |  |  | 0.13 |
| Female |  | 36(16.22%) | 48(21.62%) |  |
| Male |  | 75(33.78%) | 63(28.38%) |  |
| Age | 222 |  |  | <0.001 |
| >41 |  | 85(38.29%) | 43(19.37%) |  |
| <=41 |  | 26(11.71%) | 68(30.63%) |  |
| IDH mutation status | 221 |  |  | <0.001 |
| Mutant |  | 14(6.33%) | 98(44.34%) |  |
| Wildtype |  | 97(43.89%) | 12(5.43%) |  |
| 1p/19q codeletion status | 219 |  |  | <0.001 |
| Codeletion |  | 1(0.46%) | 49(22.37%) |  |
| Non-codeletion |  | 109(49.77%) | 60(27.40%) |  |

**Supplementary Table S3** Characteristics of patients between high- and low-risk groups in CGGA693 cohort.

| Characteristics | N | high (N=202) | low (N=202) | *P* value |
| --- | --- | --- | --- | --- |
| Grade | 404 |  |  | <0.001 |
| WHO II |  | 35(8.66%) | 95(23.51%) |  |
| WHO III |  | 55(13.61%) | 86(21.29%) |  |
| WHO IV |  | 112(27.72%) | 21(5.20%) |  |
| Gender | 404 |  |  | 0.84 |
| Female |  | 88(21.78%) | 85(21.04%) |  |
| Male |  | 114(28.22%) | 117(28.96%) |  |
| Age | 404 |  |  | <0.001 |
| >41 |  | 129(31.93%) | 97(24.01%) |  |
| <=41 |  | 73(18.07%) | 105(25.99%) |  |
| IDH mutation status | 367 |  |  | <0.001 |
| Mutant |  | 41(11.17%) | 156(42.51%) |  |
| Wildtype |  | 142(38.69%) | 28(7.63%) |  |
| 1p/19q codeletion status | 347 |  |  | <0.001 |
| Codeletion |  | 17(4.90%) | 68(19.60%) |  |
| Non-codeletion |  | 169(48.70%) | 93(26.80%) |  |

**Supplementary Table S4** The correlation coefficients between the drugs and risk score in public cohorts.

| Drugs | TCGA-GBMLGG | CGGA325 | CGGA693 |
| --- | --- | --- | --- |
| A.443654 | -0.7635 | -0.7338 | -0.5725 |
| Rapamycin | -0.7789 | -0.7669 | -0.6063 |
| Pazopanib | -0.7669 | -0.7339 | -0.5702 |
| PHA.665752 | -0.7640 | -0.6909 | -0.5412 |
| A.770041 | -0.7332 | -0.7677 | -0.4911 |
| SB.216763 | -0.2666 | -0.5486 | -0.4964 |
| GSK269962A | -0.6161 | -0.7666 | -0.5153 |
| Z.LLNle.CHO | -0.7467 | -0.6604 | -0.5068 |
| Docetaxel | -0.7138 | -0.7698 | -0.4704 |
| CGP.60474 | -0.7885 | -0.7449 | -0.4352 |
| JNJ.26854165 | -0.4743 | -0.7309 | -0.4204 |
| GNF.2 | -0.6340 | -0.5710 | -0.4339 |
| Bryostatin.1 | -0.7011 | -0.6486 | -0.4846 |
| KIN001.135 | -0.1025 | -0.4271 | -0.4447 |
| Bortezomib | -0.7476 | -0.6941 | -0.4576 |
| NVP.TAE684 | -0.4556 | -0.6694 | -0.4425 |
| NVP.BEZ235 | -0.4903 | -0.6284 | -0.4291 |
| Cyclopamine | -0.7159 | -0.7051 | -0.4555 |
| FTI.277 | -0.5273 | -0.6496 | -0.4513 |
| Temsirolimus | -0.5993 | -0.5990 | -0.3814 |
| Parthenolide | -0.7865 | -0.7076 | -0.4271 |
| BI.2536 | -0.6542 | -0.6927 | -0.4476 |
| XMD8.85 | -0.7435 | -0.7095 | -0.4445 |
| PD.0325901 | -0.6185 | -0.4665 | -0.4104 |
| JW.7.52.1 | -0.7344 | -0.6442 | -0.4074 |
| Roscovitine | -0.6434 | -0.6226 | -0.4291 |
| CCT018159 | -0.4211 | -0.5040 | -0.4237 |
| NU.7441 | -0.4952 | -0.4152 | -0.3774 |
| RDEA119 | -0.6739 | -0.4842 | -0.4090 |
| Sorafenib | -0.6455 | -0.6471 | -0.4000 |
| Cisplatin | -0.5242 | -0.6289 | -0.3736 |
| Tipifarnib | -0.4912 | -0.6252 | -0.4190 |
| Metformin | -0.4404 | -0.5112 | -0.3477 |
| GW843682X | -0.6695 | -0.4747 | -0.3662 |
| Salubrinal | -0.6604 | -0.6447 | -0.3630 |
| BMS.509744 | -0.5734 | -0.5457 | -0.3690 |
| S.Trityl.L.cysteine | -0.6518 | -0.4765 | -0.3345 |
| X17.AAG | -0.7807 | -0.6943 | -0.3134 |
| WZ.1.84 | -0.6082 | -0.6766 | -0.3066 |
| AUY922 | -0.5125 | -0.4184 | -0.3349 |
| MG.132 | -0.5404 | -0.5758 | -0.3530 |
| Dasatinib | -0.7458 | -0.6034 | -0.2962 |
| Vinblastine | -0.6233 | -0.3638 | -0.3220 |
| KU.55933 | -0.6478 | -0.5481 | -0.3065 |
| CMK | -0.5243 | -0.1416 | -0.3012 |
| MK.2206 | -0.4586 | -0.5505 | -0.2868 |
| TW.37 | -0.6737 | -0.5623 | -0.2817 |
| Paclitaxel | -0.7307 | -0.5324 | -0.2758 |
| AP.24534 | -0.5597 | -0.5079 | -0.2610 |
| MS.275 | -0.7156 | -0.4412 | -0.2553 |
| AZ628 | -0.6025 | -0.3683 | -0.2862 |
| CGP.082996 | -0.7571 | -0.6640 | -0.2368 |
| Gemcitabine | -0.4979 | -0.3219 | -0.2643 |
| RO.3306 | -0.5092 | -0.4618 | -0.2548 |
| Midostaurin | -0.5686 | -0.5385 | -0.2150 |
| BMS.536924 | -0.6584 | -0.4194 | -0.2016 |
| Obatoclax.Mesylate | -0.5204 | -0.3786 | -0.2671 |
| AZD7762 | -0.3394 | -0.4021 | -0.1598 |
| VX.680 | -0.7015 | -0.4016 | -0.1558 |
| PF.02341066 | -0.5859 | -0.1549 | -0.1821 |
| WH.4.023 | -0.7530 | -0.5476 | -0.1309 |
| CEP.701 | -0.3212 | -0.2277 | -0.1984 |
| Embelin | -0.4096 | -0.5897 | -0.1460 |
| WO2009093972 | -0.2981 | -0.4404 | -0.1042 |

**Supplementary Table S5** The connectivity scores of 17 potential drugs validated in cMap.

| Drugs | Cancer cell lines | Mechanism of action | False discovery rate (log10) | Connectivity score |
| --- | --- | --- | --- | --- |
| A.443654 | SKBR3 | AKT inhibitor | 1.0789 | -1.4103 |
| BI.2536 | MCF7 | PLK inhibitor | 0.8885 | -1.2781 |
| BI.2536 | U2OS | PLK inhibitor | 0.7049 | -1.1841 |
| BI.2536 | LNCAP | PLK inhibitor | 0.0459 | -0.7545 |
| BI.2536 | SW480 | PLK inhibitor | 0.0176 | -0.6904 |
| BI.2536 | U937 | PLK inhibitor | 0.0041 | -0.6148 |
| BMS.509744 | HA1E | ITK inhibitor | 0.0466 | -0.7569 |
| bortezomib | U937 | NFKB inhibitor\|Proteasome inhibitor | 0.2124 | -0.9228 |
| CGP.60474 | JURKAT | CDK inhibitor | 0.0424 | -0.7478 |
| CGP.60474 | ASC | CDK inhibitor | 0.0058 | -0.6291 |
| cyclopamine | U2OS | Smoothened receptor antagonist | 0.0306 | -0.7249 |
| cyclopamine | MCF7 | Smoothened receptor antagonist | 0.0204 | -0.6992 |
| docetaxel | SKMEL5 | Tubulin inhibitor | 0.79 | -1.2154 |
| docetaxel | THP1 | Tubulin inhibitor | 0.194 | -0.9084 |
| docetaxel | A375 | Tubulin inhibitor | 0.0716 | -0.7946 |
| docetaxel | HBL1 | Tubulin inhibitor | 0.0129 | -0.6775 |
| GNF.2 | CD34 | Bcr-Abl inhibitor | 0.9811 | -1.3239 |
| GNF.2 | SKL | Bcr-Abl inhibitor | 0.4108 | -1.0447 |
| GNF.2 | HA1E | Bcr-Abl inhibitor | 0.0315 | -0.7273 |
| GNF.2 | HELA | Bcr-Abl inhibitor | 0.0022 | -0.5929 |
| KU.55933 | MCF10A | ATM kinase inhibitor | 0.9356 | -1.2987 |
| KU.55933 | A549 | ATM kinase inhibitor | 0.1019 | -0.8294 |
| metformin | HA1E | Insulin sensitizer | 1.0789 | -1.4322 |
| metformin | U2OS | Insulin sensitizer | 0.5317 | -1.1001 |
| metformin | MCF7 | Insulin sensitizer | 0.323 | -0.9933 |
| metformin | HELA | Insulin sensitizer | 0.2828 | -0.9686 |
| metformin | A375 | Insulin sensitizer | 0.0859 | -0.8129 |
| MG.132 | A549 | Proteasome inhibitor | 0.0189 | -0.6961 |
| NVP.BEZ235 | OCILY19 | MTOR inhibitor\|PI3K inhibitor | 0.297 | -0.977 |
| NVP.BEZ235 | VCAP | MTOR inhibitor\|PI3K inhibitor | 0.2876 | -0.9733 |
| NVP.BEZ235 | NALM6 | MTOR inhibitor\|PI3K inhibitor | 0.255 | -0.9519 |
| NVP.BEZ235 | NPC | MTOR inhibitor\|PI3K inhibitor | 0.2264 | -0.9334 |
| NVP.BEZ235 | SW480 | MTOR inhibitor\|PI3K inhibitor | 0.191 | -0.9054 |
| NVP.BEZ235 | U937 | MTOR inhibitor\|PI3K inhibitor | 0.1313 | -0.8601 |
| NVP.BEZ235 | MDAMB231 | MTOR inhibitor\|PI3K inhibitor | 0.0482 | -0.7601 |
| parthenolide | HCC515 | NFKB inhibitor | 0.4972 | -1.0868 |
| parthenolide | PHH | NFKB inhibitor | 0.3335 | -1.0022 |
| pazopanib | HUVEC | VEGFR inhibitor\|KIT inhibitor\|PDGFR inhibitor | 0.9186 | -1.293 |
| pazopanib | NCIH2073 | VEGFR inhibitor\|KIT inhibitor\|PDGFR inhibitor | 0.24 | -0.9428 |
| pazopanib | MCF10A | VEGFR inhibitor\|KIT inhibitor\|PDGFR inhibitor | 0.113 | -0.84 |
| pazopanib | PC3 | VEGFR inhibitor\|KIT inhibitor\|PDGFR inhibitor | 0.0283 | -0.72 |
| PD.0325901 | HA1E | MEK inhibitor\|MAP kinase inhibitor\|Protein kinase inhibitor | 0.3953 | -1.0355 |
| temsirolimus | HELA | MTOR inhibitor | 1.0789 | -1.5003 |
| tipifarnib | HCC515 | Farnesyltransferase inhibitor | 0.6876 | -1.1786 |
| tipifarnib | AGS | Farnesyltransferase inhibitor | 0.0633 | -0.7842 |
